# Altered Proteomic Profile of Exosomes Secreted from Vero Cells Infected with Porcine Epidemic Diarrhea Virus

**DOI:** 10.3390/v15081640

**Published:** 2023-07-27

**Authors:** Xuehuai Shen, Lei Yin, Shuangshuang Xu, Jieru Wang, Dongdong Yin, Ruihong Zhao, Xiaocheng Pan, Yin Dai, Hongyan Hou, Xueli Zhou, Xiaomiao Hu

**Affiliations:** 1 Livestock and Poultry Epidemic Diseases Research Center of Anhui Province, Institute of Animal Husbandry and Veterinary Science, Anhui Academy of Agricultural Science, Hefei 230031, China; xuehuaishen1986@126.com (X.S.); yinlei1989@yeah.net (L.Y.); 15655091239@163.com (S.X.); wangjr0317@163.com (J.W.); yindd160@163.com (D.Y.); zrhkdy@aliyun.com (R.Z.); daiyin2020@163.com (Y.D.); houyan1296@163.com (H.H.); zxli69@163.com (X.Z.); huxiaomiao66@163.com (X.H.); 2 Anhui Province Key Laboratory of Livestock and Poultry Product Safety Engineering, Hefei 230031, China; 3 College of Veterinary Medicine, Nanjing Agricultural University, Nanjing 210095, China

**Keywords:** PEDV, Vero cells, exosomes, proteomics, cellular regulatory pathway

## Abstract

Porcine epidemic diarrhea virus (PEDV) infection causes severe diarrhea in pigs and can be fatal in newborn piglets. Exosomes are extracellular vesicles secreted by cells that transfer biologically active proteins, lipids, and RNA to neighboring or distant cells. Herein, the morphology, particle size, and secretion of exosomes derived from a control and PEDV-infected group are examined, followed by a proteomic analysis of the exosomes. The results show that the exosomes secreted from the Vero cells had a typical cup–shaped structure. The average particle size of the exosomes from the PEDV-infected group was 112.4 nm, whereas that from the control group was 150.8 nm. The exosome density analysis and characteristic protein determination revealed that the content of exosomes in the PEDV-infected group was significantly higher than that in the control group. The quantitative proteomics assays revealed 544 differentially expressed proteins (DEPs) in the PEDV-infected group’s exosomes compared with those in the controls, with 236 upregulated and 308 downregulated proteins. The DEPs were closely associated with cellular regulatory pathways, such as the phosphatidylinositol–4,5–bisphosphate 3–kinase (PI3K)–protein kinase B (Akt) signaling pathway, extracellular matrix–receptor interaction, focal adhesion, and cytoskeletal regulation. These findings provide the basis for further investigation of the pathogenic mechanisms of PEDV and the discovery of novel antiviral targets.

## 1. Introduction

Porcine epidemic diarrhea virus (PEDV) is the causative pathogen of porcine epidemic diarrhea (PED), and its infection of piglets causes severe diarrhea, vomiting, and dehydration, with mortality rates of up to 100% in newborn piglets [1,2]. PEDV, belonging to the genus *Alphacoronavirus* in the family *Coronaviridae*, is an enveloped virus with a single-stranded positive-strand RNA genome. The PEDV genome comprises approximately 28 kb and encodes four structural proteins, namely the spike glycoprotein, envelope protein, membrane glycoprotein, and nucleocapsid protein, as well as an accessory protein open reading frame 3 (ORF3), and sixteen nonstructural proteins [3]. The first identification of PEDV occurred in Europe in 1978, followed by an outbreak of a mutant strain in China in 2010. Subsequently, North America experienced its first reported epidemic of PEDV in 2013. Presently, PEDV has become a global epidemic, resulting in substantial economic losses in the pig industry [4].

Exosomes are extracellular vesicles secreted by various eukaryotic cells, which are characterized by a single membrane and diameters ranging from 30 to 150 nm. The content, or cargo, of exosomes consists of lipids, nucleic acids, and proteins. Exosomes are especially enriched in transmembrane proteins, lipid-anchored membrane proteins, peripherally associated membrane proteins, and soluble proteins [5]. Growing evidence suggests that exosomes play crucial roles in a range of physiological processes, such as intercellular communication, signal transduction, coagulation, and most importantly, immune responses [6,7]. Studies have found that exosomes have a critical function in the process of the viral infection of cells. On one hand, these vesicles can contain all or part of the genetic material and proteins of the virus, which promotes the infection of other cells [8,9]. On the other hand, exosomes have the capability to transfer antiviral molecules that are induced by host immune responses, which can subsequently inhibit virus replication [10]. Bedford et al. found that during influenza virus infection, exosomes released into the respiratory tract carry virus antigens and trigger lung inflammation. They further demonstrated that the surface of these vesicles contains attachment factors for the influenza virus, which have the ability to neutralize the influenza virus and prevent virus binding and entry into target cells [11]. The composition of cellular exosomes (nucleic acids or proteins) changes after the virus infects the cell. For instance, in the case of foot-and-mouth disease virus (FMDV), full-length genomic RNA and partial viral protein were detected in exosomes isolated from FMDV-infected PK–15 cells, which can transmit infection to naive PK–15 cells and suckling mice [12]. Zhang et al. found that the grass carp reovirus infection of CIK cells resulted in changes in the content of 761 proteins in the cellular exosomes, which are involved in various cellular metabolic processes such as translation, peptide biosynthesis, and metabolism [13].

Studies have found that exosomes are involved in the infection and proliferation process of PEDV. Chen et al. reported that the levels of complements C3, C6, and complement factor B (CFB) were significantly decreased in the serum exosomes of PEDV-infected piglets and that the exosomes from infected piglets restricted the PEDV infection of LLC–PK1 cells [14]. Yin et al. found that PEDV infection could alter the levels of microRNAs (miRNAs) involved in cyclic AMP (cAMP), Hippo, transforming growth factor beta (TGF–beta), hypoxia inducible factor 1 (HIF–1), forkhead box O (FoxO), mitogen-activated protein kinase (MAPK), and Ras signaling pathways in host cell exosomes [15]. Zhao et al. found that the abundance of miRNA-328-3p was significantly downregulated in the exosomes of PEDV–infected Vero E6 cells, and this miRNA was found to target and repress TJP3 (encoding tight junction protein 3, also known as ZO–3) to inhibit PEDV infection [16]. However, the role and mechanism of host–cell–derived exosomes during PEDV infection and proliferation are still not fully understood.

In this study, we aim to isolate and characterize exosomes from PEDV–infected and uninfected Vero cells, and to examine the cellular exosome morphology, particle size, and secretory concentration. Furthermore, the proteomics of cellular exosomes are analyzed using tandem mass tag (TMT)–labeled liquid chromatography–mass spectrometry (LC–MS) to determine the differences between the exosomes from infected and uninfected cells. This study provides a foundation for further investigations of the role of exosomes in PEDV infection and proliferation and offers new insights for the prevention and control of PEDV.

## 2. Materials and Methods

### 2.1. Cell Culture and PEDV Strain

Vero cells (CVCCCL28) were obtained from the National Center for Veterinary Culture Collection (Beijing, China). The cells were cultured in Dulbecco’s Modified Eagle’s Medium (DMEM) supplemented with 10% fetal bovine serum (FBS) and 1% penicillin-streptomycin, obtained from Gibco BRL (Grand Island, NY, USA) and HyClone (Logan, UT, USA), respectively. PEDV CV777 strain (GenBank: KT323979.1) was donated by the Institute of Veterinary Medicine, Jiangsu Academy of Agricultural Sciences.

### 2.2. Exosome Isolation and Purification

Vero cells were grown in 25 cm^2^ cell culture flasks and infected with CV777 at a multiplicity of infection (MOI) of 1.0 for 24 h (PEDV–infected group). Control cells (control group) were treated with an equivalent amount of culture medium. Each group was replicated three times. Cell supernatant exosome extraction was performed using an exosome concentration kit and an exosome purification kit (Rengen Biosciences Ltd., Liaoning, China). The brief procedure was as follows: First, the cell culture supernatant was added to a 50 mL centrifuge tube, followed by the addition of 4 mL of binding buffer. The mixture was inverted, and 1.6 mL of binding resin was added. The mixture was then inverted again and incubated at room temperature for 15 min. After incubation, the mixture was centrifuged at 1500× *g* for 3 min at room temperature. The collected resin was transferred to a 15 mL purification column and allowed to stand for 2 min. The column was then centrifuged at 2000× *g* for 2 min at room temperature, and the solution from the collection tube was discarded. The concentrated resin was washed twice with 2 mL of washing solution and centrifuged at 3000× *g* for 2 min. The exosome eluate was added to the concentrated resin and allowed to stand for 5 min. The mixture was then centrifuged at 3000× *g* for 2 min at room temperature to obtain the concentrated exosome solution. The purified Vero cell exosomes were obtained by adding the concentrated exosome solution to a Super EV purification column (an ultra–pure size exclusion column) and eluting stepwise using phosphate-buffered saline (PBS). The purified exosomes were stored at −80 °C.

### 2.3. Nanoparticle Tracking Analysis (NTA)

The concentration and particle size of the extracted Vero cell exosomes were determined using a Zeta View Pmx110 Nanoparticle Tracking Analysis system (Particle Metrix, Inning am Ammersee, Germany), and the results were analyzed using ZetaView (version 8.05.14 SP7) software. The instrument was calibrated using polystyrene microspheres (100 nm).

### 2.4. Transmission Electron Microscopy (TEM)

To visualize the morphology of exosomes, an exosome suspension was mixed with a 0.2% paraformaldehyde suspension and applied onto a formvar-coated copper grid. The sample was then stained with 1% uranyl acetate in aqueous water for 2 min, followed by filtering off the excess liquid. TEM was then performed using an FEI microscope (FEI, Hillsboro, OR, USA) to examine the sample.

### 2.5. Western Blot Analysis of Exosomal Proteins

Exosome samples (20 μL) were placed in spiking buffer and denatured by heating at 97 °C for 10 min. The denatured proteins were separated using 12% sodium dodecyl sulfate polyacrylamide gel electrophoresis (SDS–PAGE) and transferred onto nitrocellulose membranes using a Bio-Rad dry blotting system (Bio–Rad, Hercules, CA, USA). The membranes were then blocked with 3% non-fat powdered milk in PBS for 1 h at 20~25 °C. Then, monoclonal antibodies against CD63, CD9, tumor susceptibility 101 (TSG101), and glyceraldehyde 3 phosphate dehydrogenase (GAPDH) (Abcam, Cambridge, UK) were diluted 1:1000 in 4% bovine serum albumin (BSA) buffer and incubated with the membranes overnight at 4 °C. The membranes were washed three times with PBST (PBS plus 0.1% Tween 20) and incubated with enzyme-labeled secondary antibodies at room temperature for 1 h. After washing the membranes with PBST three times, the chemiluminescence substrate was added, and the immunoreactive protein bands were detected using Tanon–5200Muiti fluorescence imaging system (Tanon, Shanghai, China).

### 2.6. Exosomal Protein Preparation

Frozen exosome samples were transferred into 1.5 mL Eppendorf tubes and lysed using 300 µL of lysis buffer supplemented with 1 mM phenylmethylsulfonyl fluoride (PMSF). The samples were further lysed using sonication. The parameters were set as 1 s/1 s intervals, 3 min time, and 80 W power. After sonication, the samples were centrifuged at 15,000× *g* for 15 min to remove insoluble particles, and this was repeated once to further exclude contaminants. The protein concentration was determined using a bicinchoninic acid (BCA) assay, and the protein solution was aliquoted and stored at −80 °C.

Next, 10 µg of proteins from each sample were acquired and separated using 12% SDS-PAGE gel. The gel was then stained using Coomassie brilliant blue (CBB) as follows: Firstly, the gel was fixed for 2 h and stained for 12 h. After staining, the gel was washed with water until the bands were visualized. Finally, the stained gel was scanned using a Tanon 1600 automatic digital gel image analysis system (Tanon, Shanghai, China).

### 2.7. Proteome Sequencing

The entire proteome was detected and analyzed via Shanghai Lu Ming Biological Technology Co. Ltd. (Shanghai, China). Briefly, the protein extract (100 µg) was mixed with the reducing buffer and incubated at 60 °C for 1 h. Then, indole acetic acid (IAA) was added to the solution and incubated in the dark for 40 min. The solution was centrifuged at 12,000 rpm for 20 min at 4 °C, and the flowthrough was discarded. After washing the samples twice using 100 µL of 300 mM triethylammonium bicarbonate (TEAB) buffer, 100 µL of TEAB containing 1 μg/μL trypsin was added for digestion at 37 °C for 12 h. The digested peptides were collected via centrifugation at 12,000 rpm for 20 min and lyophilized. For TMT labeling, the lyophilized sample was resuspended in 100 μL of 200 mM TEAB. At room temperature, acetonitrile was added to dissolve the TMT reagent and mixed to obtain TMT labeling reagent. Then, 41 μL of the TMT labeling reagent was added to 40 μL of samples, mixed, and incubated for 1 h at room temperature. Finally, 8 µL of 5% hydroxylamine was added to each sample and incubated for 15 min to terminate the reaction. The labeled peptide solutions were lyophilized and stored at −80 °C.

Reversed phase (RP) separation was performed on an 1100 HPLC System (Agilent, CA, USA) using an Agilent Zorbax Extend RP column (5 μm, 150 mm × 2.1 mm). Mobile phases A (2% acetonitrile in high-performance liquid chromatography (HPLC) water) and B (98% acetonitrile in HPLC water) were used for the RP gradient. Tryptic peptides were separated at a fluent flow rate of 300 μL/min and monitored at 210 nm and 280 nm. The separated peptides were lyophilized for mass spectrometry. All analyses were performed using a Q-Exactive mass spectrometer (Thermo Fisher Scientific, Waltham, MA, USA) equipped with a Nanospray Flex source (Thermo Fisher Scientific). Samples were loaded onto and separated using a C18 column (15 cm × 75 µm) on an EASY–nLC TM 1200 system (Thermo Fisher Scientific). Full MS scans were acquired in the mass range of 300~1600 *m*/*z* with a mass resolution of 70,000, and the automated gain control (AGC) target value was set at 1e6. The ten most intense peaks in the MS spectrum were fragmented with higher-energy collisional dissociation (HCD) with a normalized collision energy (NCE) of 32. MS/MS spectra were obtained with a resolution of 17,500, an AGC target of 2e5, and a maximum injection time of 80 ms. The Q–E dynamic exclusion was set at 30.0 s and run under positive mode.

### 2.8. Database Search

Proteome Discoverer v.2.4 (Thermo Fisher Scientific) was used to thoroughly search all of the Q Exactive raw data against the sample protein database. The database search was performed using Trypsin digestion specificity. Alkylation on cysteine was considered as a fixed modification during the database searching. For the protein quantification, the TMT labeling method was selected. The global false discovery rate (FDR) was set to 0.01, and protein groups considered for quantification required at least 1 peptide. The differentially expressed proteins (DEPs) were screened using a standard of fold change (FC) > 1.5 or < 0.667, and *p*-value < 0.05. All DEPs were mapped to three main categories of Gene Ontology (GO) terms for annotation, namely cellular component, molecular function, and biological process, which were obtained from the GO consortium website (http://www.ebi.ac.uk/GOA/, accessed on 17 December 2022). Kyoto Encyclopedia of Genes and Genomes (KEGG) pathway enrichment analysis was also performed using DAVID 6.7 [17,18]. Protein–protein interaction (PPI) networks were constructed using the Search Tool for the Retrieval of Interacting Genes/Proteins (STRING) database [19] (http://string.embl.de/, accessed on 8 January 2023) and further visualized using Cytoscape software [20].

### 2.9. Statistical Analysis

All statistical analyses were performed using SPSS 16.0 statistical software (IBM Corp., NY, USA). Data are expressed as the mean ± SD. In this study, independent samples *t*-test was used for comparisons between groups. *p* < 0.05 was considered to be statistically significant.

## 3. Results

### 3.1. Characterization and Secretion of Exosomes from Control and PEDV–Infected Vero Cells

**Figure 1:**
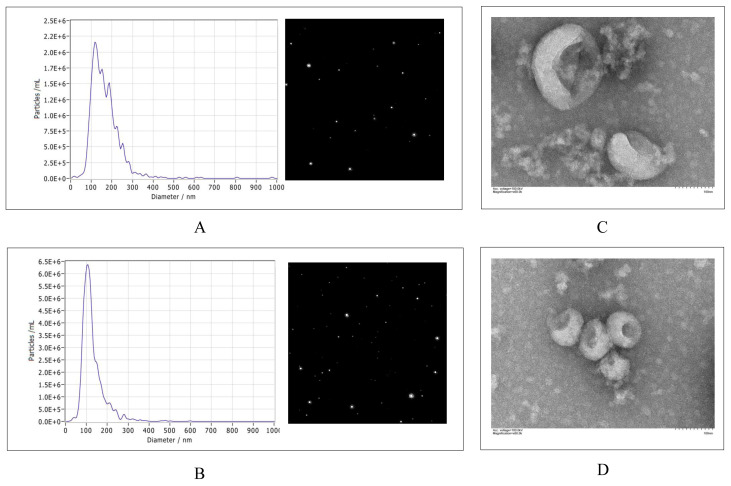
Characterization of Vero–cell–derived exosomes from PEDV–infected group and control group. (**A**) Particle size and quantification from control cells. (**B**) Particle size and quantification of PEDV–infected cells. (**C**) Morphology of exosomes from control group observed via TEM. (**D**) Morphology of exosomes from PEDV–infected group. Magnification = ×60.0 K; scale bars, 100 nm. PEDV, porcine epidemic diarrhea virus; TEM, transmission electron microscopy.

The morphology and size of the isolated Vero cell exosomes were characterized using NTA and TEM. The NTA measurements showed that the mean particle size of the exosomes of the Vero cells in the control group was 150.8 nm (Figure 1A), and the mean particle size of the exosomes in the PEDV-infected group was 112.4 nm (Figure 1B). The TEM analysis showed that the Vero cell exosomes from the control and PEDV-infected groups had a typical cup–shaped structure (Figure 1C,D).

**Figure 2:**
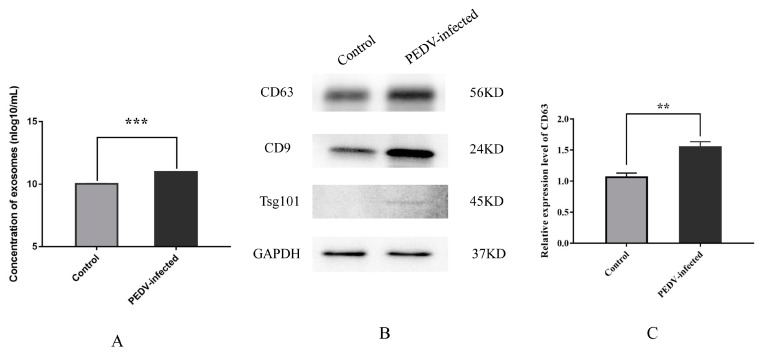
Effect of PEDV infection on the secretion of exosomes from Vero cells. (**A**) The concentration of exosomes from control and PEDV–infected groups. (**B**) Western blot detection of exosomal signature proteins CD63, CD9, and TSG101. (**C**) Relative expression of exosomal protein CD63 from control and PEDV–infected groups. Note: ** *p* < 0.01, *** *p* < 0.001. TSG101, tumor susceptibility 101; GAPDH, glyceraldehyde–3–phosphate dehydrogenase.

The NTA assay data indicate that the average concentration of exosomes in the control group was 1.07 × 10^10^ particles/mL, whereas in the PEDV–infected group, it was significantly higher at 9.37 × 10^10^ particles/mL (*p* < 0.001) (Figure 2A). The Western blot analysis for the levels of exosomal signature proteins CD63, CD9, and TSG101 (Figure 2B) showed that the relative levels of CD63 was significantly higher in the PEDV-infected group than in the control group (*p* < 0.01) (Figure 2C). These findings suggest that PEDV infection increases the content of exosomes in Vero cell culture medium.

### 3.2. Identification of Exosomal Proteins from Vero Cells

To comprehensively assess the effect of PEDV infection on the levels of proteins in exosomes secreted by Vero cells, the proteomic profiles of the exosomes were characterized using TMT labeling LC–MS/MS. In this study, 32,635 peptides and 5001 protein groups were identified with an FDR of less than 0.01. These proteins were screened for confidence using a score sequest HT of > 0 and a unique peptide of ≥1. A total of 4586 credible proteins were screened, and the information on the identified credible proteins is presented in Supplementary Table S1. The principal component analysis (PCA) of the credible proteins showed significant differences in the PCA of the Vero cell exosome proteins between the control and PEDV–infected groups (Figure S1A). The sample correlation analysis of the credible proteins showed a high inter–sample correlation and intra–group sample reproducibility (Figure S1B). The sample-level clustering analysis of the credible proteins showed that the control and PEDV–infected groups clustered in different branches, respectively (Figure S1C). The molecular weights of the identified credible proteins were mainly distributed in the range of 10 to100 kDa (Figure S1D).

### 3.3. Analysis of Differentially Expressed Proteins in Exosomes from Control and PEDV–Infected Groups

**Figure 3:**
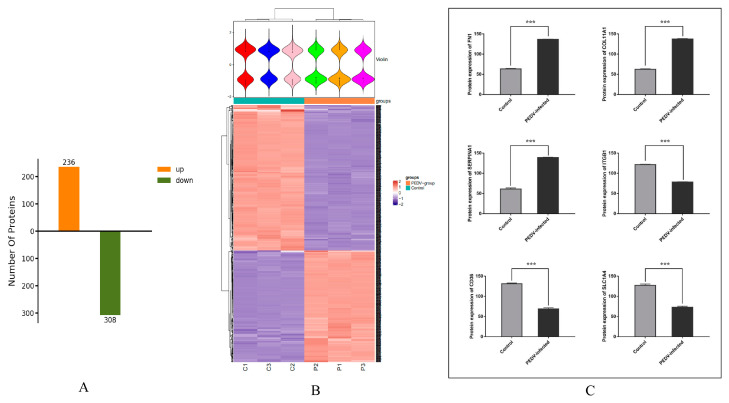
DEP analysis of exosomes from control and PEDV–infected groups. (**A**) The identified DEPs in the exosomes of PEDV–infected group compared with those in control group. (**B**) Heat map of comparative intergroup clustering analysis of DEPs. (**C**) Statistical analysis of protein expression levels of FN1, COL11A1, SERPINA1, ITGB1, CD36, and SLC1A4. Note: *** *p* < 0.001.

According to the screening criteria (FC > 1.5 and *p*-value < 0.05), 544 DEPs were identified from the exosomal protein analysis, among which 236 proteins were upregulated and 308 proteins were downregulated (Figure 3A; Supplementary Table S2). The cluster heat map analysis showed significant clustering of the DEPs between the control and PEDV-infected groups (Figure 3B). Among the DEPs, fibronectin 1 (FN1), serine protease inhibitor (SERPIN) family proteins (SERPINA1, SERPINA5, SERPINE2, SERPINF2), and collagen family proteins (COL4A1, COL4A2, COL5A1, COL5A2, COL6A2, COL7A1) were significantly upregulated, while integrin beta-1 (ITGB1) protein, tetraspanin protein family (CD36, CD44, CD151, CD58), and solute carrier family proteins (SLC1A4, SLC4A4, SLC16A1, SLC34A1) were significantly downregulated in the exosomes from the PEDV-infected cells (Figure 3C). These significant DEPs might be involved in the infection and proliferation processes of PEDV in host cells.

### 3.4. GO and KEGG Pathway Annotations of the DEPs

**Figure 4:**
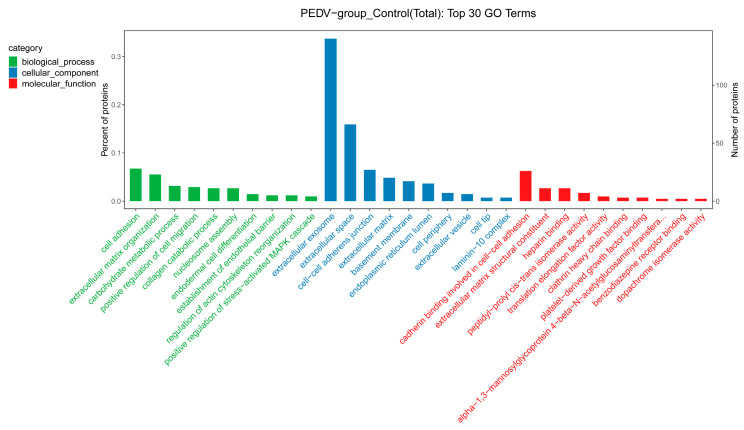
GO annotation analysis of the DEPs identified in the exosomes isolated from control and PEDV–infected groups.

**Figure 5:**
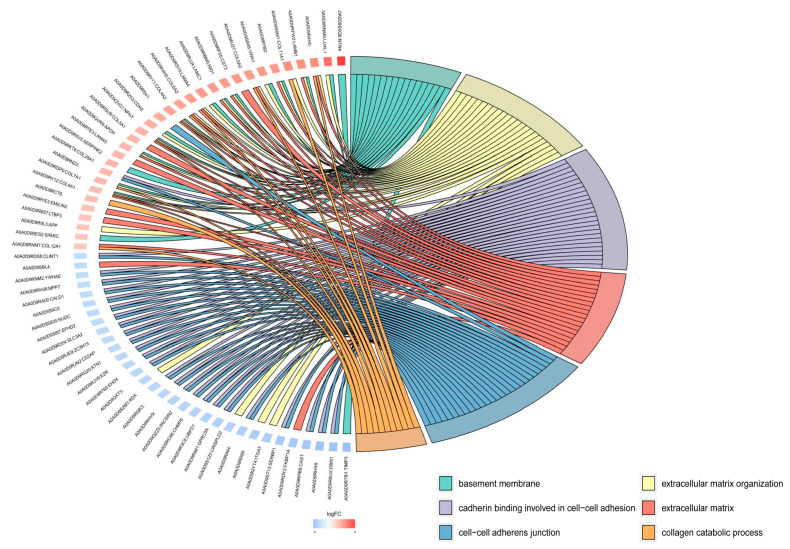
GO enrichment analysis chord diagram.

The GO enrichment analysis was used to further analyze the cellular functions of the DEPs in the Vero cell exosomes. The results show that the GO terms were enriched into three categories involving the biological process, cellular component, and molecular function. Among the top 30 enriched GO terms, cell adhesion, extracellular matrix organization, and carbohydrate metabolic process were highly enriched in the biological process category. The cellular component category mainly focuses on extracellular exosome, extracellular space, and cell–cell adherens junction. Cadherin binding is involved in cell–cell adhesion, extracellular matrix structural constituent, and heparin binding, being particularly enriched in molecular function (Figure 4). From the GO enrichment analysis chord diagram, the DEPs with upregulated expression were mainly enriched in extracellular matrix organization, extracellular matrix, and collagen catabolic process, while those with downregulated expression were mainly enriched in cadherin binding involved in cell–cell adhesion, cell–cell adherens junction, and basement membrane (Figure 5). These results suggest that the exosomal DEPs from the control and PEDV-infected groups are involved in cell adhesion, metabolism, and extracellular matrix composition.

**Figure 6:**
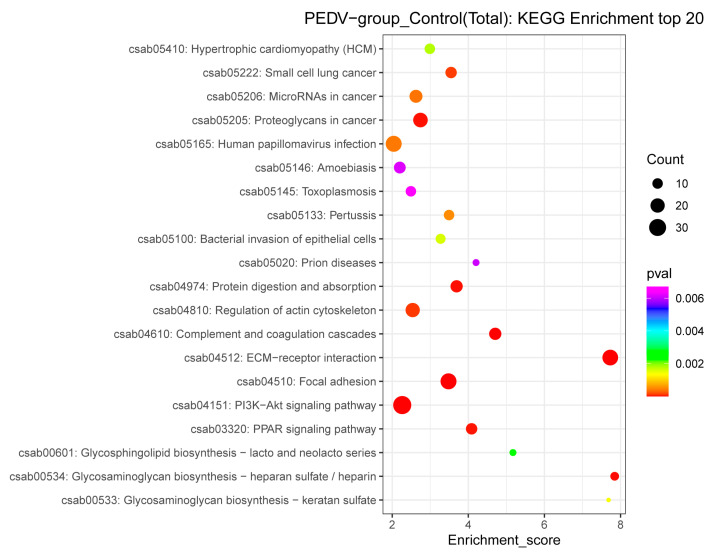
KEGG pathway analysis of the DEPs identified in the exosomes isolated from control and PEDV–infected groups.

The KEGG analysis of the DEPs showed that they are involved in a variety of life activities, and many of the proteins were annotated into the PI3K–Akt signaling pathway, focal adhesion, extracellular matrix (ECM)–receptor interaction pathway, and regulation of the actin cytoskeleton (Figure 6). Thirty-five exosomal DEPs were enriched in the PI3K–Akt signaling pathway (e.g., ITGB1, YWHAE, CDC37, and ITGA3), 26 DEPs were enriched in focal adhesion (e.g., LAMC1, RAP1B, FN1, and COL1A1), 25 DEPs were enriched in the ECM–receptor interaction pathway (e.g., CD36, HSPG2, and SPP1), and 20 DEPs were enriched in actin cytoskeleton regulation (e.g., MSN, EZR, and RDX). These results suggest that exosomes play a crucial role in cell signaling, and that many DEPs are associated with cellular immunity, cytoskeleton, and adhesion-related pathways.

### 3.5. PPI Network Analysis of the DEPs

**Figure 7:**
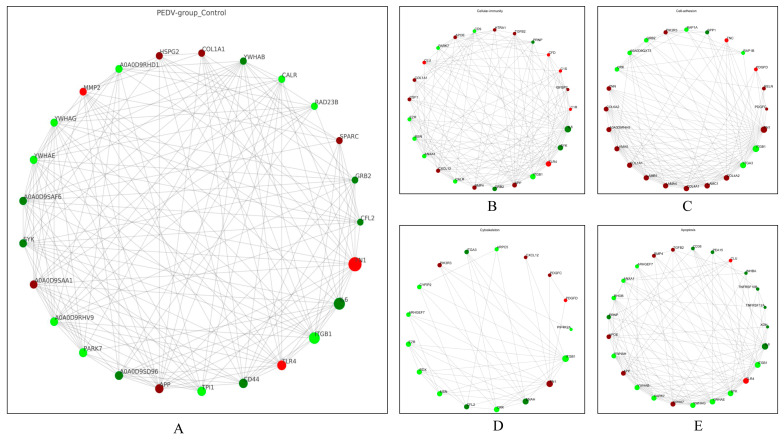
PPI network analysis of the DEPs identified in the exosomes isolated from control and PEDV–infected groups. (**A**) PPI interaction network analysis of the top 25 protein interactions among the DEPs. (**B**) PPI interaction network analysis of DEPs enriched in cellular immune responses. (**C**) PPI interaction network analysis of DEPs enriched in cell adhesion. (**D**) PPI interaction network analysis of DEPs enriched in the cytoskeleton. (**E**) PPI interaction network analysis of DEPs enriched in apoptosis. The dots in the figure represent DEPs, red represents upregulated DEPs, and green represents downregulated DEPs; the size of the dots represents the level of connectivity, with larger dots indicating higher connectivity. The straight lines represent the proteins that are correlated with each other.

In this study, the top 25 PPIs among the DEPs were analyzed to map the PPI interactions profile. The results show that 8 of the 25 DEPs were upregulated proteins, and 17 were downregulated proteins (Figure 7A). The DEPs associated with cellular immunity, cell adhesion, cytoskeleton, and cell death in the exosomes were analyzed for PPIs; those related to cellular immunity include 14 upregulated proteins (e.g., TLR4, APP, BMP4, CXCL12, CSF1, COL1A1) and 11 downregulated proteins (e.g., IL6, SYK, ITGB1, CALR, MSN, EZR) (Figure 7B), and those related to cell adhesion include 16 upregulated proteins (e.g., FN1, collagen family proteins, laminin family proteins, TNN, PIK3R3, TNC) and 8 downregulated proteins (e.g., ITGB1, ITGA3, CRK, GRB2, RAP1A, SPP1) (Figure 7C). ITGB1, ITGA3, MSN, and ENAH are upregulated proteins associated with the cytoskeleton, while FN, PIK3R3, and CXCL12 are downregulated proteins (Figure 7D); the levels of ITGB1, IL6, YWHAG, YWHAB, YWHAE, and YWHAH, which are involved in the regulation of cell apoptosis, were all decreased in the exosomes of the PEDV-infected group (Figure 7E). It is worth noting that some proteins, such as ITGB1, ITGA3, IL6, and CD44 (downregulated), and FN1, PIK3R3, TLR4, and COL1A1 (upregulated), are involved in various cell biological processes. These findings reveal that PEDV infection in Vero cells leads to significant alterations in the levels of several proteins with important biological functions in the exosomes.

## 4. Discussion

Although PEDV has been a global epidemic for many years and has caused serious economic losses in the pig industry, the pathogenic mechanism of PEDV infection in host cells still needs to be further investigated from different perspectives due to the high genetic heterogeneity of PEDV [21]. Exosomes are extracellular vesicles that are secreted by cells and play a crucial role in intercellular communication. They transfer biologically active proteins, lipids, and RNAs to neighboring or distant cells, thereby performing an important biological function [5]. However, the research on the biological functions of exosomes during the virus infection of host cells is still in its infancy [22,23]. Currently, an increasing number of researchers have focused on the role and mechanisms of host–cell–derived exosomes in PEDV infection. In this study, Vero cells are used as a research model due to their high susceptibility to PEDV infection, and are widely applied for PEDV virus isolation, vaccine preparation, and pathogenic mechanism studies [15,24,25]. Porcine intestinal epithelial cells (IECs) are generally considered an ideal cellular model for studying PEDV pathogenic mechanisms since they are the host target cells for PEDV infection. However, the low replication efficiency of PEDV in porcine intestinal cell lines limits their application [26]. It was found that PEDV infection exhibits similar mechanisms in both Vero cells and IECs. Cong et al. found that pAPN mediates the infection and release of PEDV in both Vero cells and IECs [27]. PEDV N protein activates the P53–DREAM pathway, inducing Vero cell and IEC cycle arrest at the S phase [26]. Additionally, integrin avB3 promotes PEDV replication in Vero E6 cells and IECs [28]. Therefore, it is representative to study the effects of PEDV infection on host cells’ exosome characteristics and proteomic profiles using Vero cells as model cells.

Increasing evidence suggests that exosomes are involved in the cellular immune response to infection with viruses and are associated with virus infection, proliferation, and transmission [29,30,31,32]. Exosomes originate from late endosomes called multivesicular bodies (MVBs), which are a component of the endocytic pathway. MVBs contain intraluminal vesicles (ILVs) of varying sizes, which fuse with the cytoplasmic membrane and release ILVs into the extracellular environment, at which point they are referred to as exosomes [7]. The ESCRT pathway is the most understood pathway for the mechanism of exosome biogenesis [33]. Additionally, the Rab GTPases family is also involved in exosome biogenesis [22], whereby Rab11, Rab27, Rab5, Rab35, and Rab7 were identified as key regulators of exosome secretion [33]. A study revealed that some viruses can manipulate the exosomal pathway for transmission, assembly, and excretion [34]. The “Trojan” exosome hypothesis was first proposed by Gould et al. [35], which suggests that the assembly and release of HIV share similarities with the biology of exosomes; HIV has evolved to exploit the exosome system and infect cells by packaging the virus [35,36]. HIV, Ebola virus, rabies virus, and herpes simplex virus 1 (HSV1) all employ distinct strategies to hijack members of the ESCRT pathway that are involved in exosome formation [37,38]. The studies revealed that HIV and HSV1 infection can induce alterations in the expression levels of host cell Rab27a, subsequently affecting exosome biogenesis [39,40]. Wang et al. found that rabies virus infection can increase the release of exosomes from host cells, and that the inhibitors of exosome secretion can significantly inhibit cellular exosome release and reduce viral proliferation [41]. These studies suggest that viral infections can significantly affect exosome biogenesis. Notably, our results reveal that PEDV can significantly alter the size and number of host cell exosomes, suggesting that this might be related to the mechanism by which PEDV hijacks Vero cell exosome formation. A previous study showed that the use of the exosome formation inhibitor GW4869 could effectively inhibit virus proliferation [13,41]. However, in our preliminary research, we found that even at low concentrations (5 μM), the exosome inhibitor GW4869 significantly reduced the activity of Vero cells; therefore, we will try to use other approaches to verify the role of host exosomes in PEDV proliferation. However, the specific mechanism still requires further investigation.

The proteomic analysis in this study showed that PEDV infection caused extensive changes to the protein levels in the Vero cell exosomes, and a total of 544 DEPs were identified. These exosomal DEPs might play important molecular signaling roles in cellular immunity and viral infection; e.g., ITGB1 is a transmembrane receptor that mediates the connection between the cell and its external environment [42], which was recently found to be a co-receptor for angiotensin-converting enzyme 2 (ACE2), enhancing the binding of novel coronavirus proteins to their receptor ACE2 [43]. The levels of SERPIN family proteins (SERPINA1, SERPINA5, SERPINE2, SERPINF2), which have a significant inhibitory effect on the proliferation of HIV, recombinant vesicular stomatitis virus (rVSV), and Zika virus (ZIKV) [44], were significantly increased in the PEDV–infected Vero cell exosomes. There are two independent studies on the effect of PEDV infection on the miRNA and protein levels in exosomes. Yin et al. found that the levels of 115 miRNAs were altered in the exosomes of PEDV–infected cells compared with those in the controls, and these differentially abundant miRNAs were associated with a significant increase in the levels of miRNAs through association with cAMP, Hippo, TGF–beta, HIF–1, FoxO, MAPK, and Ras signaling pathways [15]. Chen et al. found that PEDV infection significantly decreased the levels of complement proteins (C3, C6, CFB) in piglet serum exosomes, demonstrating that serum exosomes from newborn piglets can significantly inhibit PEDV infection, and that this inhibitory effect is closely related to complement [14]. In addition, Zhao et al. discovered that miRNA–328–3p is significantly downregulated in the exosomes from PEDV-infected Vero E6 cells. Furthermore, miRNA–328–3p can inhibit PEDV infection by targeting and suppressing TJP3 in receptor cells [16]. These findings indicate that investigating the alterations in exosome composition during PEDV infection can provide clues to the pathogenic mechanisms of PEDV and identify potential antiviral targets.

Exosomes act as mediators of intercellular crosstalk in metabolism, accurately reflect changes in the cellular microenvironment, and play crucial roles in information transmission, microenvironment modulation, and immune regulation [34,45,46,47]. The present study further analyzed the enriched GO categories of the DEPs and showed that many proteins were enriched in cellular biological processes such as cell adhesion, extracellular matrix organization, and carbohydrate metabolism, indicating that PEDV infection not only leads to abnormal levels of exosomal proteins associated with cellular material metabolism, but also has significant effects on the cell structure and extracellular matrix homeostasis. Moreover, the exosomal DEPs in the present study involve multiple cell signaling pathways such as the PI3K/Akt signaling pathway, focal adhesion, extracellular matrix (ECM)—receptor interaction pathway, and are also involved in the regulation of the actin cytoskeleton. The PI3K–Akt signaling pathway is a crucial intracellular signaling pathway that plays a significant role in promoting cell proliferation and metabolism, and certain viruses can exploit this signaling pathway to facilitate their own proliferation [48,49]. Herein, we revealed that 35 exosome-derived DEPs were enriched in the PI3K/Akt signaling pathway, which also represented the highest enrichment in the KEGG analysis of the DEPs. Previous studies on the effect of PEDV infection on the PI3K/Akt signaling pathway in host cells were conducted. Shen et al. found that PEDV infection significantly suppressed the expression of key molecules in the PI3K/Akt signaling pathway in IPEC–J2 cells [50]. Lin et al. found that PEDV infection suppressed the protein synthesis of IPEC–J2 cells via the downregulation of the PI3K–AKT/mTOR signaling pathways [51]. Further studies revealed that non-structural protein 6 of PEDV induces autophagy through the PI3K/Akt/mTOR axis to promote virus replication [52]. These findings indicate that PEDV infection induces significant alterations in the expression levels of regulatory molecules in the PI3K/Akt signaling pathway of the host cells. Therefore, we hypothesized that changes in the exosomal protein levels induced by PEDV infection might play an important role in intercellular transmission and regulation of the PI3K/Akt signaling pathway.

Focal-adhesion-based cell–extracellular matrix interactions are essential for cell anchoring and cell migration, and plays a crucial role in development, immunity, and disease [53,54,55]. Research has revealed that herpes simplex virus 1 (HSV–1) envelop proteins (pUL7 and pUL51), localize to focal adhesions on the cytoplasmic membrane, and maintain the attachment of infected cells to their surrounding environment by modulating the activity of focal adhesion complexes [56]. Ramsauer et al. found that the equine papillomavirus type 2 (EcPV2) infection of genital organs can cause changes in gene expression, with many DEGs being enriched in ECM–receptor interactions and focal adhesion pathways [57]. In the present study, we found that many DEPs were enriched in the focal adhesion (26 DEPs) and ECM–receptor interaction (25 DEPs) pathways. Currently, no studies have reported the association of PEDV infection with host cell focal adhesion. However, there was an interesting study on ECM–related signaling and PEDV infection, which found extensive ECM remodeling in PEDV–infected intestinal epithelial cells. Moreover, the transcript levels of two genes closely related to the ECM pathway (CD44 and SERPINE1) in PEDV-infected intestinal epithelial cells were significantly and positively correlated with the extent of PEDV replication. CD44 enhanced the expression of antiviral cytokines (such as IL–6, IL–18, and the antimicrobial peptide β-defensin 1) by activating NF–κB signaling. In contrast, PAI–1 (encoded by SERPINE1) promoted progeny virus release during PEDV infection [58]. Notably, we identified a significant decrease in the CD44 and IL6 protein levels in the exosomes of the PEDV-infected Vero cells, while the levels of SERPIN family proteins (SERPINA1, SERPINA5, SERPINE2, SERPINF2) increased significantly. These findings suggest that PEDV might inhibit the antiviral response of cells and promote viral release through the ECM-related signaling pathway.

The cytoskeleton includes microfilaments, microtubules, and intermediate fibers [59]. Viral infection alters the normal composition of the cell, affecting the cytoskeletal structure to optimize virus entry, replication, and virion production [60,61]. Zhao et al. demonstrated that infection with PEDV can compromise the integrity of the IPEC–J2 cell barrier and also affect the remodeling of cellular microfilaments [62]. Hou et al. demonstrated that microtubules are involved in PEDV infection and can influence PEDV fusion and accumulation in the perinuclear region, and they used single virus tracking techniques to dynamically observe different types of intracellular PEDV transport through microtubules [63]. Our study revealed that PEDV infection led to changes in the levels of cytoskeleton regulatory proteins in the exosomes, further confirming the involvement of cytoskeletal structures in the PEDV infection of host cells.

In particular, we identified two integrin proteins, ITGB1 and integrin subunit alpha 3 (ITGA3), which were significantly downregulated in the PEDV-infected cell-derived exosomes. These proteins are involved in various signaling pathways, including cell immune response, focal adhesion, and cellular cytoskeleton. However, their involvement in the PEDV infection of host cells has not been reported. ITGB1 acts as an ECM receptor, and recent studies have revealed that it interacts with the SARS-CoV-2 cell receptor ACE2 and assists ACE2 in mediating SARS-CoV-2 infection and entry into human cells; either reducing or blocking ITGB1 can inhibit the efficiency of SARS-CoV-2 infection [43]. In addition, ITGB1 is a key receptor required for human cytomegalovirus (HCMV) proliferation [64]. ITGA3 is an integrin located on the cell membrane that functions as a cell surface adhesion molecule, and its expression is associated with cancer metastasis [65]. A study revealed that HIV-1 infection leads to significant alterations in the gene expression profile of macrophages. Among these changes, one of the most prominent altered genes is ITGA3, the upregulation of which facilitates viral budding and release [66]. Zeng et al. found that during the infection of Vero E6 cells with porcine acute diarrhea syndrome coronavirus (SADS–CoV), autophagy was induced through the regulation of the Akt/mTOR pathway, which promoted viral replication. Moreover, it was discovered that ITGA3 exerts an effective antiviral effect by inhibiting cellular autophagy [67]. Therefore, we speculated that ITGB1 and ITGA3 might play important regulatory roles in the process of PEDV infection in host cells. However, the specific functions and regulatory mechanisms still require further research.

## 5. Conclusions

In summary, we observed significant alterations in the average particle size and secretion concentration of exosomes from the PEDV–infected group. Furthermore, using TMT-labeled LC-MS/MS, we quantitatively analyzed the differential protein compositions of the exosomes and identified 544 DEPs. These DEPs are involved in various cellular processes, such as cell adhesion, extracellular matrix organization, and carbohydrate metabolism, and are closely associated with cellular regulatory pathways including the PI3K–Akt signaling pathway, ECM–receptor interaction, focal adhesion, and cytoskeleton regulation. These findings will provide a basis for investigating the pathogenic mechanism of PEDV and discovering new antiviral targets.

## Data Availability

All data generated or analyzed during this study are included in this published article (and Supplementary Materials).

## Supplementary Materials

The following supporting information can be downloaded at https://www.mdpi.com/article/10.3390/v15081640/s1, Figure S1: The credible protein analysis and data quality control in Vero cell exosomes from control and PEDV–infected groups. (A) Principal component analysis (PCA) of credible proteins. (B) Sample correlation analysis of credible proteins. (C) Sample–level clustering analysis of the credible proteins. (D) Statistical results of the molecular weights of the credible proteins; Table S1: The detailed information of the credible proteins identified in the exosomes of Vero cells; Table S2: Information on DEPs in exosomes from control and PEDV–infected groups.
