# Supplementary material for: Altered Proteomic Profile of Exosomes Secreted from Vero Cells Infected with Porcine Epidemic Diarrhea Virus
Source: Viruses. 2023 Jul 27;15(8):1640. doi: 10.3390/v15081640 (PMC10459195; doi:10.3390/v15081640)
Supplement: Supplementary file 1 [file viruses-15-01640-s001.zip › Supplementary Files/Supplementary Table S2.docx]

Supplementary Table S2. Information on DEPs in exosomes from control and PEDV − infected groups

| Accession | protein Name | Protein expression level | | | | | | *P*−value | FC |
| --- | --- | --- | --- | --- | --- | --- | --- | --- | --- |
|  |  | PEDV −infected group | | | Control group | | |  |  |
|  |  | P1 | P2 | P3 | C1 | C2 | C3 |  |  |
| A0A0D9RHQ0 | MSN | 78.4 | 76.9 | 77.7 | 123.1 | 121.8 | 122.0 | 1.862E-07 | 0.635 |
| A0A0D9RIY5 | ABCB1 | 78.7 | 77.5 | 78.7 | 123.4 | 119.7 | 121.9 | 2.917E-06 | 0.644 |
| A0A0D9RJV9 | EZR | 79.1 | 77.6 | 78.2 | 124.0 | 120.3 | 120.7 | 4.129E-06 | 0.644 |
| A0A0D9R740 | ANXA1 | 79.0 | 78.8 | 79.0 | 122.5 | 120.0 | 120.8 | 5.683E-07 | 0.652 |
| A0A0D9RG78 | ITGB1 | 79.2 | 77.5 | 77.9 | 122.6 | 120.9 | 122.0 | 4.316E-07 | 0.642 |
| A0A0D9R760 | EHD4 | 77.8 | 78.3 | 78.0 | 122.7 | 121.4 | 121.8 | 4.591E-08 | 0.640 |
| A0A0D9RTU4 | / | 77.1 | 75.5 | 76.0 | 124.1 | 123.7 | 123.6 | 7.106E-08 | 0.616 |
| A0A0D9SCB4 | BASP1 | 75.1 | 74.1 | 72.6 | 127.4 | 124.5 | 126.4 | 1.264E-06 | 0.586 |
| A0A0D9SAF6 | / | 66.9 | 66.8 | 67.2 | 134.3 | 132.2 | 132.6 | 5.789E-08 | 0.503 |
| A0A0D9R3L1 | STIP1 | 70.4 | 69.1 | 69.0 | 131.4 | 129.9 | 130.3 | 7.068E-08 | 0.532 |
| A0A0D9RHV9 | / | 75.5 | 77.4 | 75.9 | 125.0 | 122.5 | 123.7 | 8.630E-07 | 0.616 |
| A0A0D9RPB8 | CAST | 68.4 | 69.6 | 69.0 | 132.3 | 130.1 | 130.6 | 1.287E-07 | 0.527 |
| A0A0D9S2M1 | RDX | 77.3 | 77.6 | 78.0 | 122.7 | 122.0 | 122.4 | 1.013E-08 | 0.634 |
| A0A0D9R6G5 | LDHB | 76.3 | 77.0 | 75.9 | 122.5 | 126.1 | 122.2 | 3.368E-06 | 0.618 |
| A0A0D9RHW7 | PABPC1 | 64.7 | 64.4 | 64.8 | 138.5 | 133.6 | 134.1 | 1.417E-06 | 0.477 |
| A0A0D9QUQ1 | EEA1 | 77.3 | 76.9 | 75.8 | 124.7 | 122.6 | 122.7 | 5.649E-07 | 0.622 |
| A0A0D9RNM2 | YWHAE | 80.8 | 78.1 | 80.0 | 121.5 | 120.5 | 119.1 | 2.749E-06 | 0.662 |
| A0A0D9R354 | SLC3A2 | 76.6 | 78.0 | 81.2 | 121.8 | 120.9 | 121.4 | 6.574E-06 | 0.648 |
| A0A0D9RCV3 | TPI1 | 74.3 | 75.2 | 73.7 | 127.1 | 125.2 | 124.4 | 6.034E-07 | 0.593 |
| A0A0D9RRZ8 | INHBA | 62.4 | 63.4 | 62.7 | 137.7 | 136.1 | 137.7 | 2.722E-08 | 0.458 |
| A0A0D9RQ20 | KTN1 | 78.3 | 79.4 | 77.4 | 122.8 | 120.3 | 121.9 | 1.285E-06 | 0.644 |
| A0A0D9S7P0 | PABPC4 | 70.9 | 70.3 | 69.4 | 131.8 | 128.6 | 128.9 | 7.207E-07 | 0.541 |
| A0A0D9RZW1 | YWHAG | 77.9 | 75.7 | 75.1 | 124.6 | 124.2 | 122.5 | 1.519E-06 | 0.616 |
| A0A0D9QY94 | / | 78.3 | 77.8 | 76.8 | 126.9 | 119.6 | 120.6 | 4.316E-05 | 0.634 |
| A0A0D9RT22 | PPIA | 63.5 | 63.5 | 63.8 | 138.3 | 135.3 | 135.6 | 1.806E-07 | 0.466 |
| A0A0D9RP06 | YWHAB | 73.0 | 71.3 | 72.5 | 127.5 | 128.2 | 127.6 | 5.772E-08 | 0.566 |
| A0A0D9R4G5 | CDC37 | 78.6 | 80.3 | 78.7 | 122.6 | 120.0 | 119.8 | 2.487E-06 | 0.656 |
| A0A0D9QYT4 | ITGA3 | 71.6 | 72.4 | 72.5 | 129.6 | 126.8 | 127.1 | 4.709E-07 | 0.565 |
| A0A0D9RFS7 | XDH | 64.5 | 64.1 | 64.0 | 137.2 | 135.5 | 134.7 | 7.325E-08 | 0.473 |
| A0A0D9R8U2 | DBN1 | 65.3 | 69.1 | 67.7 | 131.9 | 133.2 | 132.7 | 6.246E-07 | 0.508 |
| A0A0D9S835 | NUDC | 78.6 | 78.2 | 79.8 | 122.3 | 120.8 | 120.3 | 6.579E-07 | 0.651 |
| A0A0D9R2H5 | CALR | 80.1 | 78.2 | 80.8 | 120.3 | 121.8 | 118.9 | 3.726E-06 | 0.662 |
| A0A0D9RIN2 | TPM1 | 68.4 | 67.8 | 68.0 | 133.6 | 130.5 | 131.7 | 2.575E-07 | 0.516 |
| A0A0D9R716 | RAB1B | 78.8 | 78.1 | 78.3 | 121.3 | 122.1 | 121.4 | 1.959E-08 | 0.645 |
| A0A0D9RHD1 | / | 80.4 | 78.2 | 79.0 | 121.4 | 120.7 | 120.4 | 5.005E-07 | 0.655 |
| A0A0D9RE47 | CYFIP2 | 78.7 | 82.2 | 76.3 | 125.5 | 118.5 | 118.7 | 1.286E-04 | 0.654 |
| A0A0D9SB26 | RAP1B | 75.9 | 79.3 | 75.1 | 124.4 | 122.9 | 122.5 | 5.062E-06 | 0.623 |
| A0A0D9QVN1 | RAB6A | 78.8 | 77.3 | 78.7 | 123.4 | 119.9 | 122.0 | 2.686E-06 | 0.643 |
| A0A0D9S6F7 | RAP1A | 76.2 | 73.3 | 75.3 | 127.5 | 124.6 | 123.2 | 5.145E-06 | 0.599 |
| A0A0D9S2A3 | CALM1 | 73.9 | 76.5 | 73.8 | 125.9 | 125.9 | 124.1 | 1.191E-06 | 0.596 |
| A0A0D9QZN3 | NME2 | 74.3 | 75.2 | 73.7 | 125.6 | 125.2 | 126.1 | 5.783E-08 | 0.592 |
| A0A0D9R827 | YWHAH | 77.5 | 75.9 | 76.9 | 124.7 | 123.0 | 122.0 | 9.034E-07 | 0.623 |
| A0A0D9RED0 | CD9 | 79.6 | 78.4 | 79.3 | 122.7 | 119.9 | 120.1 | 1.743E-06 | 0.654 |
| A0A0D9R0N8 | ST13 | 79.8 | 78.7 | 79.0 | 122.3 | 118.9 | 121.4 | 2.578E-06 | 0.655 |
| A0A0D9S8I7 | EFHD2 | 76.5 | 79.8 | 79.6 | 121.3 | 120.8 | 121.9 | 2.772E-06 | 0.648 |
| A0A0D9R0F7 | TPM4 | 76.1 | 73.6 | 75.0 | 125.2 | 125.6 | 124.6 | 3.475E-07 | 0.599 |
| A0A0D9R4Q8 | CPNE8 | 50.0 | 52.4 | 51.4 | 150.3 | 147.4 | 148.5 | 9.548E-08 | 0.345 |
| A0A0D9RT26 | MARCKS | 69.1 | 69.1 | 66.8 | 135.1 | 129.7 | 130.1 | 4.829E-06 | 0.519 |
| A0A0D9RAH6 | CTTN | 63.5 | 64.3 | 63.7 | 138.1 | 135.2 | 135.2 | 2.155E-07 | 0.469 |
| A0A0D9RH60 | RAB18 | 73.7 | 73.2 | 74.9 | 128.3 | 125.4 | 124.4 | 2.134E-06 | 0.587 |
| A0A0D9R9G1 | / | 78.1 | 76.7 | 78.2 | 123.7 | 121.3 | 122.1 | 8.038E-07 | 0.635 |
| A0A0D9S713 | SERBP1 | 70.4 | 69.5 | 71.4 | 128.2 | 129.6 | 131.0 | 4.452E-07 | 0.543 |
| A0A0D9RQC0 | MDH1 | 77.2 | 76.6 | 75.1 | 123.4 | 122.9 | 124.7 | 5.465E-07 | 0.617 |
| A0A0D9RJ41 | SRI | 74.4 | 76.7 | 74.1 | 124.3 | 127.1 | 123.4 | 3.542E-06 | 0.601 |
| A0A0D9RY84 | NAPG | 75.2 | 75.7 | 73.9 | 125.3 | 124.4 | 125.5 | 1.535E-07 | 0.599 |
| A0A0D9RE60 | / | 72.1 | 75.3 | 73.2 | 128.3 | 124.7 | 126.3 | 2.946E-06 | 0.582 |
| A0A0D9RSY4 | CFL2 | 66.5 | 66.2 | 66.1 | 135.1 | 131.8 | 134.2 | 2.808E-07 | 0.496 |
| A0A0D9R7D9 | PPA1 | 77.9 | 77.8 | 77.9 | 123.0 | 120.1 | 123.3 | 1.691E-06 | 0.638 |
| A0A0D9RWY0 | UCHL1 | 73.3 | 72.2 | 73.6 | 126.8 | 126.7 | 127.4 | 3.713E-08 | 0.575 |
| A0A0D9RQK3 | / | 79.6 | 76.5 | 75.8 | 126.0 | 121.4 | 120.7 | 2.373E-05 | 0.630 |
| A0A0D9S3H8 | / | 79.8 | 79.8 | 79.8 | 121.0 | 119.3 | 120.3 | 1.332E-07 | 0.664 |
| A0A0D9S6C6 | SLC16A1 | 70.6 | 71.9 | 72.4 | 130.8 | 124.2 | 130.1 | 1.250E-05 | 0.558 |
| A0A0D9S3T7 | IGSF8 | 70.3 | 68.0 | 67.5 | 134.3 | 129.7 | 130.2 | 3.155E-06 | 0.522 |
| A0A0D9QZW8 | KRT3 | 73.0 | 73.5 | 77.6 | 126.0 | 128.4 | 121.5 | 3.477E-05 | 0.596 |
| A0A0D9SBF5 | / | 71.0 | 68.2 | 68.8 | 130.3 | 130.0 | 131.8 | 4.519E-07 | 0.530 |
| A0A0D9RE73 | CLTA | 73.2 | 75.1 | 72.9 | 127.9 | 123.6 | 127.3 | 4.081E-06 | 0.584 |
| A0A0D9S8R1 | PARK7 | 76.0 | 76.6 | 76.8 | 124.8 | 122.4 | 123.4 | 3.589E-07 | 0.619 |
| A0A0D9RIS4 | RAB8B | 63.2 | 70.7 | 75.4 | 134.0 | 128.2 | 128.5 | 1.141E-04 | 0.536 |
| A0A0D9RAH2 | PLIN2 | 63.5 | 65.1 | 62.8 | 137.9 | 134.3 | 136.3 | 5.231E-07 | 0.469 |
| A0A0D9QZT6 | EIF4B | 62.9 | 63.1 | 63.7 | 139.5 | 134.5 | 136.3 | 9.866E-07 | 0.462 |
| A0A0D9R9F1 | PLAU | 64.4 | 63.8 | 62.8 | 139.6 | 133.5 | 135.8 | 2.454E-06 | 0.467 |
| A0A0D9QWU6 | SRGAP1 | 62.5 | 68.8 | 61.6 | 133.2 | 142.9 | 131.0 | 7.708E-05 | 0.474 |
| A0A0D9RJN2 | CD2AP | 78.2 | 77.5 | 79.7 | 123.9 | 122.0 | 118.7 | 1.286E-05 | 0.646 |
| A0A0D9RBE8 | SLC4A7 | 72.7 | 71.8 | 69.8 | 130.8 | 127.8 | 127.1 | 2.293E-06 | 0.556 |
| A0A0D9R091 | GOT1 | 78.4 | 73.5 | 78.0 | 124.2 | 123.0 | 122.8 | 8.846E-06 | 0.621 |
| A0A0D9REY3 | ANP32B | 71.0 | 72.4 | 72.7 | 129.9 | 125.5 | 128.6 | 2.382E-06 | 0.563 |
| A0A0D9RWT3 | GCC2 | 78.6 | 83.4 | 77.6 | 118.0 | 120.8 | 121.6 | 4.331E-05 | 0.665 |
| A0A0D9REN3 | / | 78.2 | 77.7 | 76.0 | 125.8 | 120.8 | 121.5 | 1.166E-05 | 0.630 |
| A0A0D9RN63 | IL6 | 65.8 | 65.0 | 64.3 | 136.2 | 133.3 | 135.4 | 2.193E-07 | 0.482 |
| A0A0D9RAG9 | CLTB | 73.1 | 77.5 | 72.4 | 124.4 | 127.2 | 125.5 | 8.815E-06 | 0.591 |
| A0A0D9QZZ5 | PACSIN2 | 74.9 | 75.6 | 75.5 | 126.0 | 124.4 | 123.7 | 2.635E-07 | 0.604 |
| A0A0D9RNF7 | CSRP1 | 63.2 | 61.5 | 63.8 | 137.4 | 136.7 | 137.4 | 5.494E-08 | 0.458 |
| A0A0D9S7Z0 | FABP3 | 77.9 | 75.8 | 76.0 | 124.4 | 123.4 | 122.6 | 6.395E-07 | 0.620 |
| A0A0D9S9T1 | RHOB | 78.8 | 79.3 | 77.5 | 122.3 | 120.8 | 121.3 | 4.099E-07 | 0.647 |
| A0A0D9S6V2 | CCN1 | 75.0 | 75.3 | 74.9 | 126.7 | 123.9 | 124.2 | 6.231E-07 | 0.601 |
| A0A0D9RHX8 | / | 68.8 | 66.9 | 66.8 | 132.9 | 131.2 | 133.3 | 2.361E-07 | 0.510 |
| A0A0D9R7B1 | TIMP3 | 69.2 | 63.8 | 63.9 | 134.6 | 134.2 | 134.2 | 2.744E-06 | 0.489 |
| A0A0D9S232 | SLC7A5 | 77.1 | 78.5 | 78.1 | 121.9 | 123.0 | 121.4 | 2.470E-07 | 0.638 |
| A0A0D9R8Y5 | / | 50.8 | 50.8 | 51.7 | 150.8 | 147.7 | 148.3 | 6.429E-08 | 0.343 |
| A0A0D9RG80 | RAD23B | 79.5 | 78.4 | 78.9 | 123.1 | 119.0 | 121.2 | 4.272E-06 | 0.652 |
| A0A0D9SBL4 | / | 80.2 | 79.3 | 80.0 | 121.2 | 118.3 | 121.1 | 2.152E-06 | 0.664 |
| A0A0D9SC15 | / | 73.6 | 73.6 | 72.7 | 126.8 | 125.9 | 127.4 | 5.781E-08 | 0.579 |
| A0A0D9S9C5 | / | 79.6 | 79.2 | 78.0 | 121.2 | 121.5 | 120.5 | 1.934E-07 | 0.652 |
| A0A0D9R304 | RPAP3 | 77.0 | 78.2 | 78.8 | 123.6 | 121.5 | 120.9 | 1.440E-06 | 0.639 |
| A0A0D9RE65 | GLIPR2 | 75.7 | 80.0 | 77.5 | 124.6 | 120.0 | 122.2 | 1.662E-05 | 0.636 |
| A0A0D9RLG0 | NUDT5 | 76.2 | 75.9 | 73.0 | 125.3 | 122.6 | 127.0 | 6.894E-06 | 0.600 |
| A0A0D9RVT9 | STX7 | 75.4 | 76.1 | 76.5 | 123.8 | 122.9 | 125.3 | 3.972E-07 | 0.613 |
| A0A0D9RLC9 | FABP4 | 77.9 | 79.1 | 75.2 | 124.4 | 122.2 | 121.2 | 7.054E-06 | 0.631 |
| A0A0D9QXQ1 | PDCD5 | 75.7 | 72.0 | 72.4 | 128.0 | 123.5 | 128.4 | 1.090E-05 | 0.579 |
| A0A0D9R881 | GPRC5A | 72.4 | 73.7 | 75.8 | 129.6 | 122.2 | 126.3 | 2.492E-05 | 0.587 |
| A0A0D9RHK1 | CD36 | 66.4 | 72.1 | 67.6 | 131.3 | 133.1 | 129.5 | 6.491E-06 | 0.523 |
| A0A0D9R6H2 | PDXK | 80.3 | 76.0 | 77.0 | 121.6 | 121.9 | 123.2 | 5.673E-06 | 0.636 |
| A0A0D9RI38 | SPAG1 | 74.1 | 74.0 | 78.8 | 122.5 | 128.2 | 122.4 | 3.996E-05 | 0.608 |
| A0A0D9RLP0 | / | 75.7 | 79.5 | 76.7 | 123.9 | 121.2 | 123.1 | 5.237E-06 | 0.630 |
| A0A0D9R7R5 | TP53I3 | 77.5 | 76.4 | 78.1 | 123.7 | 122.8 | 121.4 | 6.878E-07 | 0.631 |
| A0A0D9R1K5 | CORO1A | 61.1 | 60.3 | 63.8 | 143.1 | 133.8 | 138.0 | 1.206E-05 | 0.446 |
| A0A0D9R464 | / | 72.4 | 72.9 | 71.7 | 130.0 | 125.2 | 127.9 | 2.674E-06 | 0.566 |
| A0A0D9R975 | MTPN | 79.5 | 77.8 | 77.7 | 122.5 | 119.6 | 123.0 | 3.619E-06 | 0.644 |
| A0A0D9R047 | MPO | 70.1 | 72.7 | 70.6 | 131.9 | 125.1 | 129.6 | 1.143E-05 | 0.552 |
| A0A0D9RHE8 | / | 72.4 | 74.7 | 74.8 | 126.6 | 125.3 | 126.1 | 4.691E-07 | 0.587 |
| A0A0D9RFJ6 | DNAJB9 | 78.9 | 79.0 | 77.7 | 119.7 | 124.1 | 120.5 | 7.074E-06 | 0.647 |
| A0A0D9S6X0 | ADGRL2 | 78.0 | 79.9 | 78.2 | 121.2 | 119.6 | 123.2 | 3.787E-06 | 0.649 |
| A0A0D9RC66 | CHMP5 | 74.5 | 75.1 | 75.9 | 127.5 | 123.2 | 123.8 | 3.815E-06 | 0.602 |
| A0A0D9RS20 | / | 71.6 | 73.0 | 72.1 | 132.0 | 124.6 | 126.7 | 1.570E-05 | 0.565 |
| A0A0D9SDY5 | / | 82.4 | 78.3 | 78.8 | 122.4 | 117.9 | 120.2 | 2.518E-05 | 0.664 |
| A0A0D9RY65 | DNAJC3 | 77.8 | 79.2 | 79.0 | 123.2 | 120.3 | 120.4 | 2.167E-06 | 0.649 |
| A0A0D9RJE9 | ZC3H15 | 79.0 | 77.7 | 79.0 | 121.2 | 120.5 | 122.6 | 5.739E-07 | 0.647 |
| A0A0D9S3U1 | NAA25 | 75.7 | 78.2 | 78.1 | 117.6 | 130.3 | 120.1 | 3.353E-04 | 0.630 |
| A0A0D9RDR7 | CD151 | 76.3 | 74.8 | 75.9 | 123.9 | 126.5 | 122.6 | 2.446E-06 | 0.609 |
| A0A0D9RCY4 | / | 71.0 | 68.9 | 69.1 | 131.6 | 128.4 | 131.1 | 9.085E-07 | 0.534 |
| A0A0D9RPZ5 | EIF1AX | 64.6 | 65.9 | 65.5 | 134.4 | 137.2 | 132.4 | 1.126E-06 | 0.485 |
| A0A0D9S4E3 | TAOK3 | 76.8 | 80.5 | 78.4 | 124.3 | 122.1 | 118.0 | 3.616E-05 | 0.647 |
| A0A0D9S1W8 | COTL1 | 71.4 | 72.7 | 70.5 | 131.9 | 125.4 | 128.1 | 8.893E-06 | 0.557 |
| A0A0D9S860 | STMN1 | 70.7 | 74.3 | 73.6 | 129.3 | 126.3 | 125.8 | 3.993E-06 | 0.573 |
| A0A0D9RVJ2 | / | 61.7 | 73.8 | 64.9 | 129.8 | 138.8 | 130.9 | 1.339E-04 | 0.502 |
| A0A0D9S4M8 | ARHGDIA | 77.8 | 76.4 | 75.9 | 124.2 | 124.9 | 120.8 | 4.695E-06 | 0.622 |
| A0A0D9RIJ2 | CIRBP | 49.5 | 48.4 | 48.4 | 149.6 | 155.7 | 148.3 | 1.548E-06 | 0.323 |
| A0A0D9S020 | EIF4H | 68.8 | 72.4 | 67.3 | 123.1 | 156.4 | 112.0 | 1.047E-02 | 0.533 |
| A0A0D9S1U7 | / | 78.6 | 77.1 | 76.3 | 123.1 | 124.4 | 120.5 | 4.420E-06 | 0.630 |
| A0A0D9S3H2 | PFDN2 | 76.4 | 75.4 | 75.1 | 126.2 | 124.0 | 122.9 | 1.273E-06 | 0.608 |
| A0A0D9RNF6 | HINT1 | 67.5 | 66.8 | 68.3 | 132.8 | 129.9 | 134.8 | 1.641E-06 | 0.510 |
| A0A0D9RUQ9 | ENAH | 64.3 | 63.3 | 68.0 | 135.6 | 132.3 | 136.4 | 3.336E-06 | 0.484 |
| A0A0D9RA00 | CALD1 | 78.2 | 78.9 | 80.0 | 123.4 | 118.3 | 121.3 | 1.164E-05 | 0.653 |
| A0A0D9RDS8 | CLINT1 | 79.8 | 80.5 | 79.6 | 123.2 | 119.3 | 117.6 | 1.833E-05 | 0.666 |
| A0A0D9QZF5 | CD44 | 67.0 | 64.7 | 67.6 | 134.9 | 133.8 | 132.1 | 6.138E-07 | 0.497 |
| A0A0D9QXT3 | / | 73.8 | 76.0 | 83.2 | 125.4 | 117.4 | 124.3 | 2.942E-04 | 0.635 |
| A0A0D9S7K7 | YBX1 | 61.0 | 64.4 | 59.6 | 139.2 | 142.3 | 133.6 | 1.244E-05 | 0.446 |
| A0A0D9RAA8 | AGPAT1 | 71.3 | 71.4 | 71.9 | 129.1 | 125.6 | 130.8 | 3.205E-06 | 0.557 |
| A0A0D9RQG3 | SLC1A4 | 69.4 | 73.5 | 75.2 | 130.6 | 124.6 | 126.9 | 2.401E-05 | 0.571 |
| A0A0D9R6F8 | BTN1A1 | 74.5 | 74.5 | 72.5 | 126.8 | 126.2 | 125.4 | 2.969E-07 | 0.585 |
| A0A0D9S850 | SH3BGRL3 | 54.3 | 56.5 | 54.3 | 145.2 | 149.5 | 140.2 | 5.486E-06 | 0.380 |
| A0A0D9RLT7 | STMN2 | 57.3 | 56.9 | 56.5 | 146.6 | 145.2 | 137.4 | 7.344E-06 | 0.398 |
| A0A0D9R612 | SPECC1 | 71.7 | 74.5 | 74.0 | 129.6 | 121.2 | 129.0 | 4.779E-05 | 0.580 |
| A0A0D9SA23 | / | 78.0 | 75.1 | 77.4 | 119.0 | 127.6 | 122.9 | 6.176E-05 | 0.624 |
| A0A0D9SAI6 | / | 67.7 | 75.0 | 73.4 | 127.7 | 129.3 | 126.9 | 1.771E-05 | 0.563 |
| A0A0D9RE22 | RAB4A | 75.2 | 75.1 | 72.9 | 125.5 | 127.7 | 123.6 | 3.360E-06 | 0.592 |
| A0A0D9SBD4 | / | 75.0 | 72.3 | 75.1 | 126.7 | 125.2 | 125.8 | 8.860E-07 | 0.589 |
| A0A0D9QUM0 | CFDP1 | 77.2 | 76.1 | 80.0 | 123.7 | 118.1 | 124.9 | 4.958E-05 | 0.636 |
| A0A0D9RUM1 | LTF | 64.1 | 67.1 | 64.4 | 137.7 | 131.7 | 135.0 | 3.908E-06 | 0.484 |
| A0A0D9R0P3 | SLC4A8 | 77.5 | 75.7 | 73.3 | 128.9 | 117.8 | 126.8 | 1.715E-04 | 0.606 |
| A0A0D9S7P4 | PPIE | 74.8 | 71.5 | 74.5 | 130.8 | 125.0 | 123.4 | 2.890E-05 | 0.582 |
| A0A0D9RU64 | EDF1 | 72.5 | 73.2 | 74.5 | 128.6 | 126.6 | 124.5 | 2.276E-06 | 0.580 |
| A0A0D9RL58 | SPNS2 | 76.6 | 81.0 | 78.9 | 121.5 | 121.1 | 120.9 | 5.028E-06 | 0.651 |
| A0A0D9SBC9 | EIF3J | 67.8 | 67.0 | 68.6 | 130.4 | 134.6 | 131.7 | 1.068E-06 | 0.513 |
| A0A0D9S7K0 | SLC2A1 | 77.0 | 76.8 | 77.5 | 124.3 | 121.1 | 123.4 | 1.226E-06 | 0.627 |
| A0A0D9RVS4 | RNASE4 | 77.8 | 77.7 | 79.1 | 119.3 | 118.9 | 127.2 | 9.115E-05 | 0.642 |
| A0A0D9QWB0 | / | 74.8 | 72.7 | 73.6 | 127.9 | 126.1 | 125.0 | 9.170E-07 | 0.583 |
| A0A0D9RW31 | CCL28 | 75.3 | 75.1 | 77.3 | 129.1 | 120.0 | 123.2 | 6.278E-05 | 0.612 |
| A0A0D9S7X8 | TXLNA | 74.7 | 80.4 | 78.0 | 120.2 | 128.0 | 118.8 | 1.739E-04 | 0.635 |
| A0A0D9RUS3 | TNFRSF10B | 59.8 | 59.2 | 58.9 | 142.4 | 141.1 | 138.6 | 2.354E-07 | 0.421 |
| A0A0D9RD07 | TNFRSF12A | 67.6 | 69.7 | 72.2 | 133.5 | 127.6 | 129.4 | 1.041E-05 | 0.536 |
| A0A0D9RDY2 | FKBP1A | 69.6 | 70.8 | 68.2 | 136.4 | 128.7 | 126.4 | 4.023E-05 | 0.533 |
| A0A0D9RRV8 | ABCB11 | 80.3 | 71.6 | 76.5 | 124.1 | 122.0 | 125.5 | 6.154E-05 | 0.615 |
| A0A0D9R3Q3 | FERMT3 | 67.5 | 67.9 | 67.2 | 133.4 | 133.1 | 130.9 | 1.479E-07 | 0.510 |
| A0A0D9SB63 | / | 73.1 | 71.4 | 72.3 | 128.3 | 128.8 | 126.1 | 5.459E-07 | 0.566 |
| A0A0D9QXH8 | GRB2 | 66.8 | 73.8 | 71.7 | 130.0 | 120.7 | 137.0 | 3.449E-04 | 0.548 |
| A0A0D9R472 | ADAM17 | 79.7 | 76.7 | 77.0 | 122.9 | 126.3 | 117.5 | 8.387E-05 | 0.636 |
| A0A0D9S986 | / | 52.0 | 52.5 | 53.3 | 149.8 | 146.1 | 146.3 | 1.868E-07 | 0.357 |
| A0A0D9RDZ9 | SH3BGRL | 77.7 | 78.1 | 78.5 | 123.5 | 120.2 | 122.0 | 1.508E-06 | 0.641 |
| A0A0D9SE02 | / | 59.0 | 59.1 | 56.3 | 142.3 | 144.0 | 139.3 | 9.066E-07 | 0.410 |
| A0A0D9R810 | PTRHD1 | 76.6 | 80.9 | 76.0 | 123.0 | 123.3 | 120.2 | 1.729E-05 | 0.637 |
| A0A0D9RJR5 | ARPC5 | 76.3 | 78.1 | 77.6 | 120.8 | 125.4 | 121.8 | 7.070E-06 | 0.630 |
| A0A0D9R1A9 | STX3 | 70.6 | 73.4 | 73.0 | 130.3 | 129.6 | 123.1 | 2.289E-05 | 0.567 |
| A0A0D9QYI0 | / | 69.2 | 59.8 | 65.4 | 137.0 | 136.2 | 132.5 | 2.116E-05 | 0.479 |
| A0A0D9SAP4 | DPM3 | 76.1 | 75.1 | 77.0 | 129.5 | 120.1 | 122.2 | 7.900E-05 | 0.614 |
| A0A0D9S7Q0 | MYCBP | 78.3 | 79.8 | 80.8 | 122.1 | 117.9 | 121.0 | 9.643E-06 | 0.662 |
| A0A0D9RAK2 | SLC6A6 | 73.2 | 76.1 | 75.1 | 125.1 | 123.1 | 127.4 | 4.749E-06 | 0.597 |
| A0A0D9R9S4 | HK3 | 55.9 | 65.7 | 60.8 | 147.1 | 139.6 | 130.8 | 1.404E-04 | 0.437 |
| A0A0D9R6X1 | HS1BP3 | 68.4 | 73.2 | 83.5 | 116.7 | 130.5 | 127.7 | 1.236E-03 | 0.600 |
| A0A0D9S2E7 | TXNRD1 | 73.7 | 72.8 | 69.7 | 138.7 | 119.2 | 125.8 | 6.744E-04 | 0.563 |
| A0A0D9RP66 | AKAP12 | 65.6 | 64.1 | 67.0 | 135.8 | 136.3 | 131.2 | 2.954E-06 | 0.488 |
| A0A0D9S252 | HMGB2 | 75.1 | 68.0 | 75.4 | 130.1 | 126.3 | 125.2 | 4.372E-05 | 0.573 |
| A0A0D9R2N9 | TRIR | 73.7 | 75.9 | 70.6 | 131.9 | 125.6 | 122.3 | 7.747E-05 | 0.580 |
| A0A0D9RJ49 | / | 71.7 | 78.1 | 79.9 | 122.9 | 125.7 | 121.7 | 7.012E-05 | 0.620 |
| A0A0D9RKF1 | PFDN1 | 78.3 | 80.3 | 73.7 | 124.3 | 123.5 | 119.8 | 4.688E-05 | 0.632 |
| A0A0D9RQ96 | TNFAIP8 | 72.3 | 72.3 | 71.1 | 129.5 | 129.1 | 125.6 | 1.725E-06 | 0.561 |
| A0A0D9R3U8 | NUFIP2 | 72.2 | 69.0 | 66.9 | 127.1 | 127.2 | 137.6 | 8.738E-05 | 0.531 |
| A0A0D9R4W0 | MEN1 | 69.9 | 72.4 | 72.6 | 130.4 | 114.6 | 140.1 | 1.622E-03 | 0.558 |
| A0A0D9RLI2 | / | 77.7 | 79.2 | 79.3 | 122.8 | 121.0 | 119.9 | 1.768E-06 | 0.649 |
| A0A0D9RU19 | PHPT1 | 73.6 | 68.9 | 67.7 | 130.6 | 129.7 | 129.6 | 5.174E-06 | 0.539 |
| A0A0D9R3C6 | UBFD1 | 74.5 | 69.0 | 80.9 | 122.3 | 126.9 | 126.3 | 1.739E-04 | 0.598 |
| A0A0D9S7W3 | KIAA1522 | 73.8 | 76.3 | 76.1 | 129.2 | 119.5 | 125.3 | 7.331E-05 | 0.605 |
| A0A0D9QXT2 | SLC4A4 | 45.4 | 48.2 | 43.4 | 137.7 | 177.0 | 148.4 | 7.749E-04 | 0.296 |
| A0A0D9S0E7 | / | 72.9 | 72.2 | 71.4 | 130.2 | 127.1 | 126.2 | 1.707E-06 | 0.565 |
| A0A0D9RS49 | F2RL1 | 76.8 | 74.4 | 72.2 | 125.1 | 121.8 | 129.7 | 4.262E-05 | 0.593 |
| A0A0D9S1P0 | FGG | 73.7 | 71.4 | 65.6 | 132.3 | 129.6 | 127.3 | 2.940E-05 | 0.541 |
| A0A0D9SD53 | / | 59.7 | 57.1 | 57.5 | 140.5 | 145.0 | 140.3 | 1.096E-06 | 0.409 |
| A0A0D9QV52 | GMFG | 54.7 | 55.1 | 56.5 | 148.1 | 140.4 | 145.2 | 2.697E-06 | 0.383 |
| A0A0D9RJ96 | PDCD10 | 77.1 | 75.1 | 81.8 | 126.7 | 118.8 | 120.5 | 1.459E-04 | 0.639 |
| A0A0D9RF48 | MIF | 81.2 | 76.6 | 78.4 | 124.8 | 119.8 | 119.2 | 4.398E-05 | 0.649 |
| A0A0D9SAE8 | / | 71.9 | 66.4 | 70.4 | 133.3 | 127.2 | 130.7 | 1.467E-05 | 0.533 |
| A0A0D9RY05 | ARHGEF7 | 71.0 | 72.6 | 79.3 | 105.6 | 161.8 | 109.7 | 4.813E-02 | 0.591 |
| A0A0D9S422 | MED25 | 79.6 | 78.1 | 81.2 | 116.0 | 126.0 | 119.0 | 1.931E-04 | 0.662 |
| A0A0D9RU65 | SLCO4A1 | 72.4 | 68.6 | 86.0 | 120.4 | 134.3 | 118.3 | 2.613E-03 | 0.609 |
| A0A0D9S1N8 | FGA | 71.7 | 75.8 | 71.6 | 126.3 | 126.2 | 128.4 | 4.160E-06 | 0.575 |
| A0A0D9RSK8 | BTF3 | 78.1 | 72.5 | 75.7 | 125.6 | 125.8 | 122.4 | 1.502E-05 | 0.605 |
| A0A0D9SD95 | / | 76.4 | 73.6 | 77.9 | 126.9 | 122.0 | 123.2 | 1.574E-05 | 0.612 |
| A0A0D9RYX4 | / | 62.5 | 68.7 | 64.8 | 139.1 | 135.1 | 129.7 | 2.930E-05 | 0.485 |
| A0A0D9RJ45 | PIP4K2A | 76.1 | 78.3 | 84.0 | 119.6 | 121.4 | 120.6 | 6.966E-05 | 0.659 |
| A0A0D9R779 | TOM1 | 75.6 | 77.1 | 79.4 | 121.9 | 123.7 | 122.2 | 3.343E-06 | 0.631 |
| A0A0D9S1N6 | FGB | 67.5 | 64.4 | 64.5 | 133.8 | 133.9 | 136.0 | 6.300E-07 | 0.486 |
| A0A0D9RKV9 | FAM107B | 71.2 | 76.0 | 72.1 | 127.1 | 124.2 | 129.4 | 1.393E-05 | 0.576 |
| A0A0D9S645 | CD58 | 71.7 | 71.9 | 74.1 | 126.9 | 125.9 | 129.4 | 1.854E-06 | 0.570 |
| A0A0D9SE59 | MYADM | 60.8 | 65.0 | 66.8 | 138.4 | 128.5 | 140.4 | 6.238E-05 | 0.473 |
| A0A0D9RXB7 | PPIL4 | 75.1 | 71.4 | 65.6 | 138.8 | 112.9 | 136.3 | 2.525E-03 | 0.547 |
| A0A0D9S062 | NCF1 | 52.3 | 49.8 | 53.1 | 149.6 | 146.8 | 148.4 | 1.869E-07 | 0.349 |
| A0A0D9SAS7 | / | 74.9 | 78.5 | 75.4 | 126.5 | 118.3 | 126.4 | 8.620E-05 | 0.616 |
| A0A0D9RSQ0 | CENPF | 74.1 | 79.8 | 78.6 | 122.2 | 119.6 | 125.8 | 5.568E-05 | 0.632 |
| A0A0D9RCL4 | / | 74.9 | 76.6 | 79.2 | 125.6 | 122.5 | 121.1 | 1.450E-05 | 0.625 |
| A0A0D9RX83 | MTMR12 | 63.2 | 61.5 | 60.7 | 142.2 | 138.5 | 133.9 | 6.954E-06 | 0.447 |
| A0A0D9RMS5 | METTL16 | 52.9 | 45.4 | 51.8 | 151.0 | 150.9 | 148.0 | 2.481E-06 | 0.334 |
| A0A0D9S3V4 | C1QTNF5 | 79.3 | 75.0 | 79.1 | 122.1 | 125.0 | 119.5 | 3.061E-05 | 0.637 |
| A0A0D9RW93 | C6 | 60.3 | 70.5 | 68.1 | 134.3 | 130.5 | 136.3 | 4.372E-05 | 0.496 |
| A0A0D9S396 | APOC3 | 74.4 | 75.8 | 80.5 | 124.3 | 119.8 | 125.2 | 4.955E-05 | 0.625 |
| A0A0D9RQC5 | ATP6V1H | 60.2 | 94.5 | 75.1 | 102.7 | 131.0 | 136.5 | 3.158E-02 | 0.621 |
| A0A0D9RYV6 | TPT1 | 56.9 | 53.8 | 58.5 | 146.7 | 136.5 | 147.7 | 2.213E-05 | 0.393 |
| A0A0D9SAW4 | / | 67.2 | 69.7 | 67.1 | 126.6 | 129.9 | 139.5 | 8.587E-05 | 0.515 |
| A0A0D9SD96 | / | 69.5 | 72.8 | 62.0 | 143.7 | 131.3 | 120.7 | 9.819E-04 | 0.516 |
| A0A0D9R912 | SLC34A1 | 69.0 | 72.3 | 68.4 | 131.0 | 128.5 | 130.9 | 2.075E-06 | 0.537 |
| A0A0D9RNL4 | CRK | 76.8 | 84.5 | 78.4 | 113.1 | 120.3 | 126.9 | 9.639E-04 | 0.665 |
| A0A0D9R0Q0 | PTPRJ | 72.8 | 73.8 | 74.8 | 125.2 | 127.1 | 126.2 | 3.207E-07 | 0.585 |
| A0A0D9RJY6 | GNG11 | 83.4 | 72.4 | 82.7 | 113.5 | 122.5 | 125.6 | 1.277E-03 | 0.660 |
| A0A0D9RCQ5 | PTPN6 | 62.2 | 66.7 | 61.7 | 137.0 | 141.1 | 131.2 | 2.434E-05 | 0.466 |
| A0A0D9RG77 | SYNPO | 83.5 | 71.8 | 78.2 | 128.3 | 123.6 | 114.7 | 1.058E-03 | 0.637 |
| A0A0D9S8N6 | DFFA | 73.2 | 75.9 | 75.7 | 119.0 | 110.6 | 145.6 | 9.062E-03 | 0.599 |
| S4SPY7 | HLA-DQB1 | 7.3 | 8.3 | 7.7 | 204.3 | 182.1 | 190.2 | 9.139E-06 | 0.040 |
| A0A0D9RZ38 | UFM1 | 80.7 | 80.7 | 77.9 | 120.4 | 119.8 | 120.4 | 1.857E-06 | 0.664 |
| A0A0D9RAQ8 | SIMC1 | 45.8 | 43.1 | 42.1 | 162.1 | 155.9 | 151.0 | 4.925E-06 | 0.279 |
| A0A0D9RXD0 | / | 47.6 | 48.3 | 49.4 | 154.3 | 151.9 | 148.7 | 4.458E-07 | 0.319 |
| A0A0D9RTH1 | EVA1A | 78.1 | 76.6 | 75.6 | 124.8 | 128.8 | 116.0 | 2.710E-04 | 0.623 |
| A0A0D9QW82 | / | 66.7 | 68.0 | 67.2 | 132.4 | 131.1 | 134.5 | 4.148E-07 | 0.507 |
| A0A0D9RGE8 | TM4SF18 | 75.4 | 77.9 | 80.5 | 124.0 | 122.5 | 119.6 | 2.303E-05 | 0.639 |
| A0A0D9R4K5 | SYK | 71.8 | 70.2 | 72.2 | 131.9 | 128.3 | 125.6 | 7.629E-06 | 0.555 |
| A0A0D9QW49 | / | 61.9 | 58.6 | 58.9 | 144.2 | 135.2 | 141.0 | 9.254E-06 | 0.427 |
| A0A0D9RJ64 | TXNDC17 | 68.3 | 66.4 | 72.9 | 132.1 | 121.9 | 138.3 | 2.818E-04 | 0.529 |
| A0A0D9R021 | LPO | 61.8 | 67.6 | 66.5 | 135.4 | 139.2 | 129.4 | 3.259E-05 | 0.485 |
| A0A0D9QWB5 | SPP1 | 56.0 | 56.8 | 54.9 | 146.6 | 143.1 | 142.6 | 3.523E-07 | 0.388 |
| A0A0D9RWA3 | RELL1 | 76.8 | 74.5 | 74.4 | 121.7 | 129.7 | 122.8 | 4.646E-05 | 0.603 |
| A0A0D9RGQ8 | LSM7 | 71.5 | 74.7 | 73.5 | 127.7 | 128.3 | 124.3 | 4.262E-06 | 0.578 |
| A0A0D9RPN6 | PUS10 | 31.9 | 32.8 | 32.4 | 170.9 | 164.9 | 167.1 | 1.764E-07 | 0.193 |
| A0A0D9RSF7 | PUDP | 80.9 | 77.3 | 79.3 | 118.1 | 126.6 | 117.9 | 1.659E-04 | 0.655 |
| A0A0D9RRX5 | MID1 | 75.2 | 75.7 | 81.2 | 121.1 | 120.0 | 126.8 | 9.213E-05 | 0.631 |
| A0A0D9RX03 | CIB1 | 77.8 | 79.7 | 77.2 | 110.6 | 114.2 | 140.4 | 9.902E-03 | 0.643 |
| A0A0D9S480 | CD5L | 72.3 | 74.9 | 72.1 | 131.7 | 124.5 | 124.6 | 2.969E-05 | 0.576 |
| A0A0D9RQ92 | NUCKS1 | 69.6 | 69.6 | 71.9 | 125.8 | 131.5 | 131.6 | 8.763E-06 | 0.543 |
| A0A0D9SBP1 | PRNP | 60.9 | 64.3 | 62.6 | 109.1 | 189.6 | 113.5 | 4.591E-02 | 0.456 |
| A0A0D9S3Q3 | PEA15 | 61.5 | 59.4 | 54.2 | 144.1 | 141.2 | 139.6 | 5.148E-06 | 0.412 |
| A0A0D9SBB7 | SPRY2 | 40.5 | 47.0 | 45.0 | 158.7 | 151.9 | 156.8 | 2.339E-06 | 0.283 |
| A0A0D9S1Z0 | CRISPLD2 | 69.6 | 71.9 | 79.1 | 128.6 | 121.8 | 129.0 | 1.378E-04 | 0.581 |
| A0A0D9QXJ2 | C11orf58 | 74.5 | 76.5 | 75.8 | 125.0 | 125.4 | 122.7 | 1.169E-06 | 0.608 |
| A0A0D9RGG2 | JPT2 | 71.2 | 73.8 | 71.0 | 127.8 | 124.1 | 132.1 | 2.283E-05 | 0.563 |
| A0A0D9RA52 | MYH3 | 62.9 | 65.1 | 66.5 | 137.4 | 129.8 | 138.3 | 1.697E-05 | 0.480 |
| A0A0D9RAI2 | / | 66.5 | 69.2 | 68.8 | 131.1 | 132.5 | 131.9 | 2.774E-07 | 0.517 |
| A0A0D9RJ98 | PCDHGA1 | 75.1 | 86.1 | 71.6 | 116.0 | 124.6 | 126.6 | 1.190E-03 | 0.634 |
| A0A0D9RYD4 | DLGAP1 | 70.6 | 69.4 | 69.5 | 130.4 | 131.7 | 128.5 | 4.611E-07 | 0.536 |
| A0A0D9RDG0 | HHIPL1 | 62.6 | 67.9 | 65.2 | 139.3 | 128.5 | 136.5 | 4.140E-05 | 0.484 |
| A0A0D9S0Y2 | SETD7 | 74.8 | 73.6 | 72.9 | 118.7 | 131.8 | 128.1 | 1.840E-04 | 0.585 |
| A0A0D9R142 | DCTPP1 | 65.8 | 75.4 | 68.1 | 129.1 | 129.9 | 131.7 | 3.550E-05 | 0.536 |
| A0A0D9RUP3 | FABP7 | 72.5 | 78.5 | 74.0 | 120.0 | 132.6 | 122.4 | 3.022E-04 | 0.600 |
| A0A0D9S3A5 | SIK3 | 69.8 | 72.2 | 62.6 | 132.9 | 133.9 | 128.6 | 4.329E-05 | 0.517 |
| A0A0D9SDV5 | / | 67.1 | 66.6 | 69.5 | 136.0 | 128.3 | 132.5 | 1.136E-05 | 0.512 |
| A0A0D9S482 | SDS | 47.0 | 51.5 | 45.0 | 152.7 | 152.2 | 151.7 | 7.201E-07 | 0.314 |
| A0A0D9RE08 | HINT2 | 78.6 | 80.3 | 78.9 | 119.4 | 119.2 | 123.6 | 1.094E-05 | 0.657 |
| A0A0D9RI26 | MAP1LC3A | 75.2 | 74.9 | 79.3 | 121.8 | 121.9 | 127.0 | 2.958E-05 | 0.619 |
| A0A0D9R7N3 | ARHGDIB | 46.2 | 58.7 | 47.2 | 146.8 | 148.2 | 152.9 | 2.379E-05 | 0.340 |
| A0A0D9RIP3 | YLPM1 | 69.3 | 76.0 | 69.5 | 125.2 | 130.8 | 129.2 | 3.292E-05 | 0.558 |
| A0A0D9R2T6 | / | 69.2 | 62.8 | 65.4 | 145.6 | 129.7 | 127.5 | 3.359E-04 | 0.490 |
| A0A0D9RXD9 | SEC11A | 54.6 | 57.5 | 45.8 | 152.3 | 142.1 | 147.7 | 3.257E-05 | 0.357 |
| A0A0D9RYB3 | MAFK | 70.0 | 84.6 | 74.8 | 128.0 | 120.7 | 121.9 | 6.334E-04 | 0.619 |
| A0A0D9RPD8 | TAX1BP1 | 76.8 | 75.4 | 74.8 | 121.8 | 124.0 | 127.2 | 8.373E-06 | 0.609 |
| A0A0D9S3M1 | / | 77.0 | 77.7 | 69.4 | 126.0 | 126.0 | 123.9 | 5.121E-05 | 0.596 |
| A0A0D9RH38 | MPP7 | 80.4 | 83.1 | 75.3 | 120.7 | 115.0 | 125.5 | 4.265E-04 | 0.661 |
| A0A0D9RHZ3 | CACNA2D1 | 74.9 | 77.3 | 68.6 | 133.1 | 113.6 | 132.4 | 1.565E-03 | 0.582 |
| A0A0D9RL01 | ZFP36L1 | 82.2 | 73.1 | 64.4 | 136.1 | 112.7 | 131.4 | 3.703E-03 | 0.578 |
| A0A0D9S9B1 | DUSP14 | 74.2 | 72.2 | 68.4 | 111.6 | 159.2 | 114.3 | 2.167E-02 | 0.558 |
| A0A0D9RZD6 | SIGLEC15 | 32.2 | 33.2 | 36.9 | 165.3 | 170.7 | 161.8 | 1.514E-06 | 0.206 |
| A0A0D9S0V2 | SCLT1 | 68.2 | 70.2 | 70.5 | 129.6 | 134.1 | 127.4 | 8.504E-06 | 0.534 |
| A0A0D9RKI3 | PON1 | 73.3 | 74.4 | 76.5 | 127.2 | 122.4 | 126.1 | 8.177E-06 | 0.597 |
| A0A0D9QWA1 | PYURF | 74.5 | 71.8 | 74.0 | 128.1 | 125.2 | 126.4 | 1.461E-06 | 0.580 |
| A0A0D9RR96 | PIGR | 58.0 | 56.2 | 68.3 | 137.7 | 144.2 | 135.6 | 6.811E-05 | 0.437 |
| A0A0D9RTV9 | CCDC25 | 75.6 | 81.1 | 79.2 | 122.6 | 120.0 | 121.4 | 1.788E-05 | 0.648 |
| A0A0D9S346 | ZBTB16 | 58.9 | 52.4 | 57.4 | 139.4 | 147.7 | 144.2 | 9.436E-06 | 0.391 |
| A0A0D9RDK5 | CIDEC | 72.2 | 72.2 | 73.8 | 128.0 | 124.9 | 128.9 | 2.075E-06 | 0.572 |
| A0A0D9QYH9 | CASP7 | 82.2 | 80.2 | 76.1 | 105.8 | 132.7 | 122.9 | 7.075E-03 | 0.660 |
| A0A0D9S2B9 | CWF19L2 | 34.1 | 29.9 | 30.1 | 173.8 | 167.2 | 165.0 | 1.322E-06 | 0.186 |
| A0A0D9R4C1 | YBEY | 79.0 | 78.1 | 81.2 | 124.5 | 117.6 | 119.6 | 5.238E-05 | 0.659 |
| A0A0D9R099 | / | 77.0 | 74.7 | 65.2 | 129.2 | 126.6 | 127.4 | 1.147E-04 | 0.566 |
| A0A0D9RF72 | CHIC1 | 75.7 | 70.3 | 88.2 | 121.8 | 111.7 | 132.3 | 5.306E-03 | 0.640 |
| A0A0D9R3W6 | CDR2 | 79.8 | 75.2 | 77.6 | 124.5 | 116.9 | 126.1 | 1.370E-04 | 0.633 |
| A0A0D9R7Q8 | ART4 | 22.6 | 23.0 | 21.3 | 182.2 | 174.5 | 176.4 | 3.252E-07 | 0.125 |
| A0A0D9RWJ1 | DAB2 | 39.5 | 41.6 | 39.6 | 159.7 | 156.8 | 162.9 | 3.738E-07 | 0.252 |
| A0A0D9RSI2 | LSM1 | 81.8 | 77.9 | 77.9 | 126.8 | 125.9 | 109.8 | 1.836E-03 | 0.655 |
| A0A0D9RPC7 | CHI3L1 | 59.1 | 55.3 | 55.4 | 146.6 | 144.9 | 138.7 | 5.637E-06 | 0.395 |
| A0A0D9RZH6 | KATNAL2 | 71.7 | 71.6 | 72.4 | 132.8 | 123.0 | 128.4 | 3.883E-05 | 0.561 |
| A0A0D9RWU0 | LMBRD2 | 73.1 | 68.2 | 65.5 | 134.1 | 130.2 | 128.8 | 2.218E-05 | 0.526 |
| A0A0D9RCR1 | SH3BP5 | 76.8 | 80.5 | 76.1 | 143.3 | 96.3 | 126.9 | 3.272E-02 | 0.637 |
| A0A0D9RT67 | MARVELD2 | 73.1 | 70.1 | 74.7 | 131.9 | 121.1 | 129.0 | 9.760E-05 | 0.570 |
| A0A0D9S693 | HIPK1 | 65.3 | 67.9 | 53.7 | 137.5 | 141.7 | 133.8 | 1.064E-04 | 0.453 |
| A0A0D9S8B1 | HSPG2 | 135.1 | 134.6 | 134.6 | 64.2 | 66.1 | 65.4 | 2.889E-08 | 2.066 |
| A0A0D9R9X4 | FN1 | 135.9 | 137.5 | 136.6 | 61.9 | 64.7 | 63.4 | 1.565E-07 | 2.158 |
| A0A0D9S8Z7 | AGRN | 141.3 | 140.6 | 140.9 | 57.7 | 59.9 | 59.6 | 3.549E-08 | 2.386 |
| A0A0D9RFW2 | LAMB1 | 138.0 | 137.3 | 138.1 | 61.7 | 62.5 | 62.6 | 3.845E-09 | 2.213 |
| A0A0D9S4D0 | RNF213 | 121.2 | 119.6 | 120.2 | 79.5 | 79.8 | 79.6 | 1.111E-07 | 1.511 |
| A0A0D9RJJ4 | LAMC1 | 134.8 | 134.4 | 134.1 | 64.5 | 66.2 | 66.0 | 2.884E-08 | 2.050 |
| A0A0D9RSY8 | LAMA4 | 133.6 | 134.0 | 134.8 | 65.0 | 66.5 | 66.2 | 3.095E-08 | 2.035 |
| A0A0D9RQS8 | VCAN | 156.5 | 154.0 | 154.6 | 43.7 | 45.6 | 45.7 | 4.018E-08 | 3.445 |
| A0A0D9RTE3 | LAMA5 | 126.1 | 126.5 | 126.9 | 73.0 | 74.3 | 73.1 | 3.933E-08 | 1.722 |
| A0A0D9S488 | LGALS3BP | 133.7 | 136.8 | 134.4 | 63.9 | 65.8 | 65.5 | 3.789E-07 | 2.074 |
| A0A0D9RKM6 | TNC | 146.2 | 141.8 | 144.7 | 54.2 | 57.0 | 56.0 | 5.340E-07 | 2.588 |
| A0A0D9S6M1 | COL11A1 | 138.7 | 136.6 | 137.4 | 61.0 | 62.9 | 63.4 | 1.554E-07 | 2.203 |
| A0A0D9R4H5 | / | 138.4 | 138.8 | 138.1 | 60.5 | 62.0 | 62.1 | 1.636E-08 | 2.250 |
| A0A0D9RNM7 | COL12A1 | 120.5 | 119.9 | 119.7 | 79.6 | 80.7 | 79.6 | 8.596E-08 | 1.501 |
| A0A0D9RBM5 | NID1 | 134.5 | 134.6 | 134.3 | 64.9 | 65.5 | 66.2 | 5.889E-09 | 2.052 |
| A0A0D9QYJ4 | HABP2 | 131.5 | 127.8 | 129.3 | 71.0 | 69.0 | 71.4 | 1.429E-06 | 1.838 |
| A0A4W3NN68 | APOE | 130.7 | 130.4 | 130.4 | 68.4 | 69.7 | 70.3 | 4.549E-08 | 1.879 |
| A0A0D9RY11 | COL4A2 | 131.9 | 132.9 | 132.3 | 66.6 | 67.5 | 68.8 | 8.272E-08 | 1.957 |
| A0A0D9RPF4 | EFEMP1 | 137.7 | 138.8 | 137.9 | 61.3 | 62.2 | 62.1 | 6.780E-09 | 2.233 |
| A0A0D9RJ27 | COL5A2 | 134.8 | 134.6 | 136.0 | 62.9 | 66.1 | 65.6 | 3.415E-07 | 2.083 |
| A0A0D9RSU9 | COL5A1 | 129.6 | 125.5 | 127.0 | 70.6 | 74.3 | 73.0 | 4.524E-06 | 1.754 |
| A0A0D9R4H0 | COL6A2 | 134.4 | 132.7 | 133.3 | 65.3 | 67.8 | 66.5 | 1.765E-07 | 2.006 |
| A0A0D9RUM4 | LOXL2 | 143.1 | 139.2 | 142.8 | 56.9 | 58.9 | 59.2 | 5.421E-07 | 2.429 |
| A0A0D9QUP8 | TENM4 | 129.2 | 127.9 | 129.4 | 70.3 | 71.3 | 71.9 | 1.044E-07 | 1.810 |
| A0A0D9QWX9 | GLG1 | 124.3 | 127.1 | 125.4 | 73.6 | 74.6 | 75.1 | 6.427E-07 | 1.687 |
| A0A0D9SCL3 | HIST1H1E | 146.1 | 142.3 | 144.1 | 54.5 | 56.8 | 56.2 | 2.774E-07 | 2.582 |
| A0A0D9R5V5 | SERPINE2 | 127.8 | 124.9 | 126.1 | 72.0 | 75.4 | 73.8 | 2.194E-06 | 1.712 |
| A0A0D9RVG0 | BMP1 | 133.1 | 133.7 | 132.2 | 65.8 | 67.2 | 68.1 | 1.288E-07 | 1.984 |
| A0A0D9RSP0 | COL7A1 | 124.7 | 125.6 | 126.4 | 73.6 | 74.9 | 74.8 | 1.514E-07 | 1.687 |
| A0A0D9QZ51 | FBLN1 | 144.1 | 138.9 | 140.2 | 59.2 | 59.9 | 57.7 | 1.077E-06 | 2.394 |
| A0A0D9RYI2 | OLFM4 | 139.8 | 137.7 | 139.6 | 59.7 | 61.5 | 61.8 | 1.246E-07 | 2.279 |
| A0A0D9RTW6 | CLU | 132.8 | 135.1 | 131.6 | 65.6 | 68.2 | 66.7 | 8.135E-07 | 1.993 |
| A0A0D9SCK7 | HIST1H2BK | 117.9 | 122.5 | 120.5 | 79.0 | 80.3 | 79.8 | 8.050E-06 | 1.509 |
| A0A0D9SCJ9 | HIST1H1B | 148.2 | 146.3 | 147.4 | 49.8 | 55.7 | 52.5 | 7.696E-07 | 2.797 |
| A0A0D9SA35 | HIST3H2BB | 121.8 | 126.2 | 123.9 | 77.3 | 75.0 | 75.8 | 4.836E-06 | 1.630 |
| A0A0D9QYL9 | IGFBP7 | 124.3 | 130.1 | 124.5 | 72.6 | 72.7 | 75.7 | 1.670E-05 | 1.714 |
| A0A0D9RZ03 | CDH2 | 127.7 | 130.0 | 130.3 | 70.2 | 72.2 | 69.7 | 8.012E-07 | 1.829 |
| A0A0D9S8P6 | CLSTN1 | 135.1 | 131.7 | 132.1 | 67.8 | 66.5 | 66.8 | 5.401E-07 | 1.984 |
| A0A0D9RF55 | CST3 | 133.9 | 134.9 | 134.8 | 64.6 | 66.6 | 65.3 | 5.215E-08 | 2.054 |
| A0A0D9S957 | PRSS23 | 130.4 | 130.1 | 129.2 | 69.8 | 70.2 | 70.1 | 9.743E-09 | 1.855 |
| A0A0D9SCL7 | HIST1H1C | 157.3 | 149.7 | 151.0 | 45.5 | 47.8 | 48.7 | 1.998E-06 | 3.225 |
| A0A0D9QX48 | HTRA1 | 129.7 | 127.7 | 129.1 | 68.7 | 72.9 | 71.8 | 2.013E-06 | 1.811 |
| A0A0D9REJ2 | SERPINA5 | 155.8 | 145.5 | 147.4 | 49.2 | 51.1 | 51.0 | 6.667E-06 | 2.966 |
| A0A0D9S2A8 | TENM3 | 123.7 | 124.6 | 123.1 | 74.5 | 77.1 | 76.9 | 9.162E-07 | 1.625 |
| A0A0D9QYW4 | COL1A1 | 123.6 | 122.1 | 122.2 | 76.6 | 78.2 | 77.3 | 2.875E-07 | 1.585 |
| A0A0D9SCL1 | HIST1H1D | 167.0 | 157.8 | 159.6 | 39.3 | 37.5 | 38.8 | 1.765E-06 | 4.190 |
| A0A0D9R7K9 | CTSF | 124.9 | 119.8 | 121.7 | 76.7 | 79.0 | 77.9 | 1.092E-05 | 1.568 |
| A0A0D9SAA1 | / | 125.8 | 116.9 | 123.1 | 77.6 | 80.9 | 75.7 | 1.343E-04 | 1.562 |
| A0A0D9RVW9 | PRPF6 | 124.7 | 126.6 | 123.1 | 75.6 | 74.4 | 75.6 | 1.384E-06 | 1.660 |
| A0A0D9S1V5 | MMP10 | 121.8 | 121.4 | 122.3 | 77.6 | 78.5 | 78.5 | 4.118E-08 | 1.558 |
| A0A0D9R8Y6 | CHRDL1 | 141.8 | 139.1 | 142.0 | 57.2 | 59.8 | 60.0 | 3.780E-07 | 2.389 |
| A0A0D9S1T7 | MMP7 | 135.2 | 135.0 | 138.4 | 62.8 | 63.3 | 65.2 | 6.644E-07 | 2.136 |
| A0A0D9R8S0 | HADHB | 119.2 | 120.8 | 120.7 | 80.1 | 79.5 | 79.7 | 1.996E-07 | 1.507 |
| A0A0D9RA05 | TCN2 | 146.6 | 146.6 | 147.1 | 51.9 | 53.6 | 54.2 | 1.977E-08 | 2.757 |
| A0A0D9RI29 | PTX3 | 130.5 | 130.5 | 127.9 | 69.0 | 72.7 | 69.4 | 2.188E-06 | 1.842 |
| A0A0D9S6Y9 | ACADM | 118.9 | 121.2 | 120.5 | 79.9 | 80.0 | 79.6 | 5.148E-07 | 1.506 |
| A0A0D9QZ20 | MMP19 | 133.6 | 129.2 | 133.4 | 67.3 | 68.2 | 68.3 | 1.647E-06 | 1.944 |
| A0A0D9RJ85 | GLUL | 130.5 | 126.7 | 127.2 | 70.8 | 72.4 | 72.5 | 1.778E-06 | 1.782 |
| A0A0D9RW48 | POLR1B | 123.0 | 121.7 | 121.9 | 77.2 | 77.8 | 78.3 | 1.075E-07 | 1.571 |
| A0A0D9RP19 | HYAL1 | 128.1 | 134.3 | 127.2 | 68.8 | 72.3 | 69.2 | 1.789E-05 | 1.853 |
| A0A0D9RHQ4 | LAS1L | 126.0 | 130.0 | 127.0 | 70.6 | 74.2 | 72.2 | 4.071E-06 | 1.765 |
| A0A0D9QWX7 | OAT | 124.7 | 123.4 | 124.8 | 75.6 | 75.7 | 75.8 | 4.591E-08 | 1.642 |
| A0A0D9RT60 | TGFB2 | 127.2 | 125.8 | 125.2 | 73.5 | 74.1 | 74.3 | 1.360E-07 | 1.704 |
| A0A0D9RFK1 | CLSTN2 | 135.4 | 134.9 | 134.4 | 64.2 | 65.4 | 65.8 | 2.503E-08 | 2.071 |
| A0A0D9R9L3 | APP | 119.3 | 120.4 | 123.6 | 79.6 | 78.7 | 78.3 | 6.144E-06 | 1.536 |
| A0A0D9QZS3 | EXT2 | 128.4 | 128.6 | 127.2 | 71.8 | 72.0 | 72.0 | 2.310E-08 | 1.780 |
| A0A0D9RN48 | SERPINF2 | 140.4 | 140.1 | 140.2 | 58.5 | 60.5 | 60.3 | 2.431E-08 | 2.346 |
| A0A0D9S1V7 | MMP1 | 131.2 | 127.1 | 129.8 | 69.2 | 72.0 | 70.7 | 2.219E-06 | 1.832 |
| A0A0D9SCM1 | HIST1H1A | 130.8 | 130.5 | 132.7 | 68.5 | 70.2 | 67.4 | 5.037E-07 | 1.912 |
| A0A0D9RR47 | DAG1 | 129.7 | 133.2 | 131.0 | 66.6 | 70.3 | 69.2 | 1.965E-06 | 1.911 |
| A0A0D9S1Q9 | PDGFC | 125.7 | 126.6 | 122.6 | 74.3 | 76.3 | 74.5 | 3.366E-06 | 1.665 |
| A0A0D9S8X6 | VWA1 | 137.4 | 133.1 | 135.6 | 62.9 | 66.6 | 64.4 | 1.753E-06 | 2.094 |
| A0A0D9RGR6 | RELN | 124.8 | 124.9 | 125.5 | 73.0 | 77.4 | 74.3 | 2.891E-06 | 1.670 |
| A0A0D9RMD6 | TIMP1 | 131.5 | 129.2 | 129.2 | 67.5 | 71.7 | 70.8 | 2.273E-06 | 1.857 |
| A0A0D9RZL0 | LIPG | 128.4 | 126.8 | 127.9 | 69.6 | 71.8 | 75.5 | 6.424E-06 | 1.766 |
| A0A0D9S381 | OLFML2B | 136.2 | 130.4 | 135.5 | 64.1 | 67.4 | 66.5 | 5.173E-06 | 2.031 |
| A0A0D9S165 | MEGF8 | 138.1 | 136.7 | 141.2 | 59.9 | 61.4 | 62.7 | 9.816E-07 | 2.261 |
| A0A0D9R473 | FSTL1 | 141.5 | 140.1 | 143.4 | 57.4 | 59.1 | 58.6 | 1.701E-07 | 2.427 |
| A0A0D9RYE4 | LFNG | 133.3 | 133.6 | 136.2 | 64.8 | 65.5 | 66.5 | 3.189E-07 | 2.048 |
| A0A0D9R9L4 | IGFBP2 | 120.2 | 121.9 | 118.6 | 78.2 | 80.4 | 80.8 | 5.436E-06 | 1.507 |
| A0A0D9SEA6 | HS3ST1 | 135.8 | 135.0 | 137.2 | 62.5 | 66.0 | 63.5 | 4.992E-07 | 2.125 |
| A0A0D9R9H6 | HS3ST3B1 | 135.5 | 133.6 | 134.7 | 62.1 | 63.6 | 70.6 | 1.332E-05 | 2.057 |
| A0A0D9S1Y3 | CPE | 140.8 | 137.0 | 137.0 | 61.1 | 61.0 | 63.0 | 7.162E-07 | 2.241 |
| A0A0D9S4P9 | NOTUM | 125.0 | 128.2 | 126.5 | 75.6 | 73.0 | 71.7 | 3.523E-06 | 1.724 |
| A0A0D9S487 | TIMP2 | 137.6 | 140.9 | 140.3 | 60.9 | 60.5 | 59.8 | 1.956E-07 | 2.311 |
| A0A0D9RY12 | COL4A1 | 122.6 | 126.1 | 124.1 | 77.1 | 74.2 | 75.8 | 3.216E-06 | 1.642 |
| A0A0D9RZQ3 | ECM1 | 126.8 | 125.0 | 128.3 | 72.0 | 76.4 | 71.4 | 8.414E-06 | 1.729 |
| A0A0D9R541 | / | 130.5 | 133.4 | 133.1 | 63.6 | 71.6 | 67.8 | 1.300E-05 | 1.956 |
| A0A0D9S589 | GBA | 123.3 | 123.0 | 123.2 | 75.8 | 76.8 | 78.0 | 2.216E-07 | 1.602 |
| A0A0D9RJX4 | PAPLN | 130.5 | 130.6 | 134.2 | 68.1 | 69.0 | 67.6 | 9.985E-07 | 1.931 |
| A0A0D9R636 | PCSK5 | 127.2 | 125.9 | 125.3 | 73.1 | 73.8 | 74.8 | 2.504E-07 | 1.707 |
| A0A0D9RNF5 | ATP6AP2 | 139.3 | 136.9 | 137.8 | 61.2 | 62.1 | 62.7 | 8.309E-08 | 2.226 |
| A0A0D9RJB0 | GOLIM4 | 124.3 | 120.1 | 122.8 | 76.8 | 79.0 | 77.1 | 5.845E-06 | 1.577 |
| A0A0D9SD49 | H1F0 | 124.1 | 123.1 | 123.5 | 75.6 | 77.8 | 75.9 | 3.791E-07 | 1.617 |
| A0A0D9RI80 | MSLN | 124.3 | 124.3 | 124.8 | 78.2 | 73.2 | 75.2 | 4.759E-06 | 1.648 |
| A0A0D9RX79 | MFGE8 | 128.5 | 127.0 | 127.3 | 72.2 | 74.7 | 70.3 | 2.163E-06 | 1.762 |
| A0A0D9SA36 | HIST3H2A | 120.1 | 125.4 | 118.4 | 76.4 | 82.2 | 77.5 | 1.025E-04 | 1.541 |
| A0A0D9QXB5 | B4GALNT1 | 128.1 | 130.3 | 125.5 | 70.8 | 73.4 | 71.8 | 3.795E-06 | 1.777 |
| A0A0D9S690 | OLFML3 | 142.5 | 128.2 | 129.9 | 68.0 | 66.7 | 64.8 | 1.295E-04 | 2.008 |
| A0A0D9SDR0 | CHST12 | 136.9 | 136.8 | 139.2 | 61.3 | 64.1 | 61.7 | 3.549E-07 | 2.207 |
| A0A0D9RES2 | SPARC | 121.1 | 121.5 | 118.8 | 80.5 | 79.9 | 78.3 | 2.769E-06 | 1.514 |
| A0A0D9R3V1 | B4GALT4 | 143.5 | 141.3 | 144.8 | 55.2 | 57.5 | 57.8 | 3.177E-07 | 2.520 |
| A0A0D9RAN3 | CRTAP | 121.6 | 120.4 | 119.2 | 78.8 | 81.8 | 78.2 | 6.362E-06 | 1.513 |
| A0A0D9QX26 | CTR9 | 121.1 | 124.4 | 120.4 | 78.6 | 76.3 | 79.2 | 8.469E-06 | 1.563 |
| A0A0D9R0V6 | PROS1 | 132.4 | 131.3 | 132.7 | 67.9 | 68.0 | 67.7 | 1.255E-08 | 1.947 |
| A0A0D9S489 | CANT1 | 129.4 | 128.9 | 129.2 | 72.0 | 69.9 | 70.5 | 8.731E-08 | 1.824 |
| A0A0D9RN23 | / | 133.0 | 120.0 | 124.2 | 72.1 | 74.5 | 76.3 | 2.148E-04 | 1.692 |
| A0A0D9R657 | LTBP3 | 122.8 | 119.7 | 122.3 | 77.9 | 77.5 | 79.8 | 3.489E-06 | 1.551 |
| A0A0D9S2X6 | CA11 | 150.2 | 149.1 | 148.7 | 50.1 | 51.3 | 50.7 | 6.535E-09 | 2.945 |
| A0A0D9QX66 | GALNT18 | 141.7 | 136.8 | 141.8 | 59.2 | 59.8 | 60.8 | 1.253E-06 | 2.338 |
| A0A0D9RFX7 | PCOLCE2 | 125.1 | 119.6 | 125.0 | 75.7 | 77.2 | 77.5 | 1.664E-05 | 1.605 |
| A0A0D9QZ39 | CRLF1 | 142.6 | 138.0 | 139.6 | 60.7 | 59.6 | 59.5 | 5.610E-07 | 2.337 |
| A0A0D9RX81 | HAPLN3 | 147.1 | 146.2 | 143.9 | 52.5 | 53.8 | 56.5 | 4.509E-07 | 2.686 |
| A0A0D9S3X5 | OAF | 127.2 | 127.8 | 127.4 | 74.0 | 71.7 | 71.9 | 2.155E-07 | 1.757 |
| A0A0D9RHK4 | CTHRC1 | 128.6 | 128.6 | 130.0 | 70.3 | 71.1 | 71.4 | 5.564E-08 | 1.820 |
| A0A0D9REQ0 | EXT1 | 140.8 | 137.3 | 139.3 | 60.8 | 61.0 | 60.8 | 1.702E-07 | 2.286 |
| A0A0D9QVG1 | NPNT | 127.2 | 127.2 | 129.9 | 70.3 | 73.4 | 71.9 | 1.552E-06 | 1.782 |
| A0A0D9S8S3 | NOL9 | 118.3 | 122.9 | 122.2 | 78.7 | 77.6 | 80.3 | 1.319E-05 | 1.536 |
| A0A0D9R035 |  | 153.6 | 159.2 | 154.9 | 43.0 | 44.6 | 44.8 | 3.901E-07 | 3.532 |
| A0A0D9SD56 | A4GALT | 123.6 | 126.3 | 122.3 | 76.7 | 73.8 | 77.3 | 7.247E-06 | 1.634 |
| A0A0D9RFH4 | TEX10 | 127.1 | 126.2 | 124.1 | 72.4 | 74.2 | 76.0 | 2.946E-06 | 1.695 |
| A0A0D9RBC2 | NIPSNAP1 | 127.4 | 122.7 | 122.5 | 76.7 | 74.9 | 75.9 | 8.738E-06 | 1.638 |
| A0A0D9RV59 | MGAT5 | 134.7 | 136.1 | 131.4 | 66.6 | 66.2 | 65.0 | 1.310E-06 | 2.033 |
| A0A0D9RZR8 | SRPX | 139.7 | 131.9 | 141.3 | 60.2 | 64.2 | 62.8 | 1.780E-05 | 2.206 |
| A0A0D9REV5 | SERPINA1 | 140.4 | 138.4 | 138.3 | 60.2 | 64.1 | 58.7 | 1.508E-06 | 2.279 |
| A0A0D9S4R3 | NAPSA | 128.8 | 119.8 | 126.7 | 77.4 | 73.1 | 74.2 | 7.562E-05 | 1.670 |
| A0A0D9S789 | CPT2 | 124.4 | 122.2 | 122.0 | 80.7 | 73.5 | 77.2 | 3.258E-05 | 1.593 |
| A0A0D9RAP9 | WNT7A | 136.0 | 129.9 | 133.0 | 69.9 | 65.3 | 66.0 | 8.368E-06 | 1.983 |
| A0A0D9RQ45 | PPIC | 139.5 | 146.8 | 133.3 | 56.1 | 60.2 | 64.2 | 6.229E-05 | 2.325 |
| A0A0D9R765 | B4GAT1 | 153.5 | 150.2 | 153.6 | 46.2 | 48.1 | 48.3 | 1.423E-07 | 3.207 |
| A0A0D9RCL7 | C1R | 137.2 | 133.4 | 137.9 | 64.1 | 63.3 | 64.1 | 8.969E-07 | 2.133 |
| A0A0D9SA92 | HIST2H2AB | 125.9 | 134.3 | 123.6 | 72.5 | 69.5 | 74.2 | 9.317E-05 | 1.775 |
| A0A0D9R7Z1 | KDM2A | 123.0 | 122.5 | 120.9 | 77.7 | 77.5 | 78.4 | 3.528E-07 | 1.568 |
| A0A0D9S7F0 | PIK3R3 | 120.0 | 120.5 | 120.4 | 72.1 | 83.6 | 83.4 | 4.363E-04 | 1.509 |
| A0A0D9R7L1 | MGAT4B | 122.4 | 123.2 | 121.2 | 76.0 | 78.9 | 78.4 | 1.977E-06 | 1.572 |
| A0A0D9S8A8 | WNT4 | 115.8 | 127.9 | 127.0 | 75.7 | 76.7 | 76.9 | 2.715E-04 | 1.617 |
| A0A0D9RC51 | C1S | 140.7 | 128.5 | 129.6 | 63.1 | 68.9 | 69.2 | 1.132E-04 | 1.982 |
| A0A0D9RUA2 | OGFR | 131.4 | 119.4 | 118.0 | 77.3 | 78.5 | 75.4 | 4.565E-04 | 1.595 |
| A0A0D9QVR9 | APOH | 131.0 | 120.8 | 128.2 | 72.8 | 73.7 | 73.5 | 6.320E-05 | 1.727 |
| A0A0D9RFV4 | MMP11 | 124.3 | 120.8 | 119.2 | 74.9 | 85.5 | 75.3 | 3.452E-04 | 1.546 |
| A0A0D9RWE8 | HS6ST1 | 128.3 | 130.1 | 128.5 | 70.5 | 70.7 | 72.0 | 1.587E-07 | 1.815 |
| A0A0D9S0B0 | TPST1 | 129.5 | 125.4 | 132.1 | 72.8 | 71.0 | 69.3 | 1.225E-05 | 1.816 |
| A0A0D9RXG4 | EPHA7 | 120.8 | 122.6 | 117.7 | 79.5 | 79.6 | 79.9 | 9.222E-06 | 1.511 |
| A0A0D9R802 | CHST3 | 123.8 | 122.2 | 124.1 | 79.7 | 73.8 | 76.4 | 1.325E-05 | 1.610 |
| A0A0D9S2T2 | ACSF3 | 124.2 | 125.1 | 121.2 | 75.4 | 76.3 | 77.8 | 4.321E-06 | 1.614 |
| A0A0D9RBU0 | ST3GAL1 | 129.7 | 118.7 | 129.4 | 67.4 | 75.4 | 79.4 | 5.079E-04 | 1.700 |
| A0A0D9R318 | TAF6L | 120.5 | 120.3 | 120.5 | 79.8 | 77.5 | 81.4 | 3.537E-06 | 1.514 |
| A0A0D9S1S1 | CLPTM1 | 126.3 | 116.4 | 124.5 | 71.9 | 80.0 | 80.9 | 4.289E-04 | 1.577 |
| A0A0D9RYE3 | EMILIN2 | 113.1 | 130.0 | 125.3 | 78.5 | 76.7 | 76.5 | 8.523E-04 | 1.590 |
| A0A0D9S8Y6 | INTS11 | 121.9 | 118.2 | 122.1 | 78.2 | 79.8 | 79.8 | 7.213E-06 | 1.523 |
| A0A0D9RIY3 | CPQ | 122.8 | 124.9 | 126.6 | 72.9 | 75.7 | 77.0 | 7.041E-06 | 1.659 |
| A0A0D9RBK8 | GTF3C2 | 137.5 | 135.7 | 143.7 | 61.0 | 60.8 | 61.3 | 5.611E-06 | 2.277 |
| A0A0D9S7R4 | GNL2 | 117.3 | 125.8 | 119.6 | 74.4 | 82.0 | 80.8 | 2.700E-04 | 1.529 |
| A0A0D9QYH8 | / | 120.0 | 127.3 | 118.9 | 75.3 | 82.0 | 76.5 | 1.911E-04 | 1.566 |
| A0A0D9RKL5 | MED6 | 121.1 | 153.7 | 130.1 | 66.5 | 67.6 | 61.0 | 2.145E-03 | 2.075 |
| A0A0D9RCN7 | NSMCE2 | 120.4 | 122.5 | 122.2 | 77.9 | 75.8 | 81.2 | 1.408E-05 | 1.554 |
| A0A0D9R4M7 | BICC1 | 127.5 | 126.5 | 129.7 | 71.6 | 74.8 | 69.9 | 5.376E-06 | 1.774 |
| A0A0D9RRG1 | CEMIP | 118.3 | 125.1 | 120.3 | 82.5 | 79.9 | 73.9 | 1.978E-04 | 1.539 |
| A0A0D9RGF6 | TM4SF1 | 131.7 | 151.4 | 115.4 | 71.6 | 62.2 | 67.6 | 3.639E-03 | 1.979 |
| A0A0D9S6M5 | EXTL2 | 133.5 | 120.2 | 137.3 | 69.8 | 69.6 | 69.6 | 3.050E-04 | 1.871 |
| A0A0D9RJT4 | CFD | 135.3 | 137.3 | 130.8 | 67.2 | 61.6 | 67.7 | 1.483E-05 | 2.053 |
| A0A0D9RJI2 | ALDH6A1 | 117.9 | 144.1 | 135.0 | 69.9 | 57.6 | 75.6 | 2.286E-03 | 1.955 |
| A0A0D9S812 | MED18 | 116.5 | 124.5 | 121.8 | 77.9 | 75.9 | 83.4 | 2.089E-04 | 1.530 |
| A0A0D9RHC8 | RC3H1 | 129.1 | 126.0 | 125.8 | 71.2 | 72.8 | 75.2 | 4.390E-06 | 1.738 |
| A0A0D9RX06 | SEMA4B | 114.6 | 139.9 | 112.4 | 74.6 | 88.3 | 70.3 | 1.262E-02 | 1.573 |
| A0A0D9S4Y7 | GLB1L3 | 133.1 | 128.0 | 132.9 | 68.1 | 66.5 | 71.5 | 9.490E-06 | 1.912 |
| A0A0D9R3I4 | REPIN1 | 127.7 | 118.7 | 116.4 | 79.7 | 75.9 | 81.6 | 3.992E-04 | 1.530 |
| A0A0D9RQR1 | BMP4 | 126.0 | 128.3 | 128.5 | 73.4 | 70.9 | 72.9 | 9.698E-07 | 1.762 |
| A0A0D9RTP8 | NOP9 | 131.0 | 131.2 | 129.5 | 66.8 | 68.4 | 73.1 | 6.364E-06 | 1.880 |
| A0A0D9SDN5 |  | 117.3 | 125.8 | 120.6 | 73.5 | 81.9 | 80.9 | 3.036E-04 | 1.539 |
| A0A0D9R872 | ANAPC16 | 135.2 | 108.6 | 120.1 | 79.7 | 79.8 | 76.7 | 5.402E-03 | 1.541 |
| A0A0D9R211 | SYNGR1 | 133.1 | 124.7 | 124.0 | 71.9 | 72.3 | 74.0 | 5.341E-05 | 1.750 |
| A0A0D9RGA0 | MRPS34 | 121.1 | 119.4 | 121.3 | 80.9 | 78.6 | 78.8 | 1.703E-06 | 1.518 |
| A0A0D9RKV8 | PAIP2 | 124.1 | 130.3 | 108.5 | 76.4 | 93.3 | 67.4 | 1.370E-02 | 1.531 |
| A0A0D9RJ71 | PKDCC | 125.2 | 116.8 | 120.1 | 84.0 | 76.8 | 77.0 | 2.609E-04 | 1.523 |
| A0A0D9QUL1 | CHST6 | 138.9 | 130.3 | 138.5 | 61.8 | 66.0 | 64.6 | 1.965E-05 | 2.119 |
| A0A0D9QZ11 | MMP2 | 145.1 | 143.8 | 148.3 | 53.5 | 56.6 | 52.8 | 8.503E-07 | 2.684 |
| A0A0D9S1R2 | APOC1 | 126.9 | 126.9 | 136.3 | 60.0 | 81.1 | 68.9 | 9.459E-04 | 1.858 |
| A0A0D9RDS1 | ATAD2 | 119.3 | 127.8 | 126.3 | 75.4 | 73.3 | 77.9 | 7.608E-05 | 1.648 |
| A0A0D9S6K2 | WDR47 | 114.4 | 121.8 | 124.6 | 83.6 | 73.1 | 82.4 | 8.399E-04 | 1.509 |
| A0A0D9SC40 | ZBTB38 | 137.8 | 136.7 | 130.5 | 58.3 | 66.5 | 70.1 | 7.338E-05 | 2.078 |
| A0A0D9RCQ9 | SQLE | 121.0 | 120.4 | 120.0 | 86.9 | 73.0 | 78.6 | 5.367E-04 | 1.515 |
| A0A0D9RU55 | VOPP1 | 141.1 | 150.8 | 137.5 | 54.7 | 53.7 | 62.3 | 5.695E-05 | 2.516 |
| A0A0D9R1I6 | KMT2D | 135.7 | 127.6 | 123.7 | 71.8 | 74.6 | 66.5 | 1.678E-04 | 1.818 |
| A0A0D9RX64 | POLG | 129.1 | 125.1 | 133.0 | 66.8 | 73.2 | 72.8 | 4.640E-05 | 1.820 |
| A0A0D9RIH9 | IFT80 | 117.9 | 126.7 | 116.1 | 81.3 | 70.4 | 87.6 | 2.515E-03 | 1.507 |
| A0A0D9RHW2 | CHTF18 | 131.7 | 131.6 | 130.7 | 68.6 | 68.0 | 69.4 | 2.742E-08 | 1.913 |
| A0A0D9RDM9 | ATP6V0C | 124.0 | 123.9 | 121.1 | 74.5 | 80.3 | 76.1 | 2.002E-05 | 1.598 |
| A0A0D9S264 | PDGFD | 148.3 | 156.0 | 159.9 | 43.1 | 32.7 | 60.0 | 2.249E-04 | 3.418 |
| A0A0D9R635 | HS6ST2 | 125.0 | 119.3 | 120.0 | 78.8 | 78.5 | 78.4 | 1.839E-05 | 1.546 |
| A0A0D9RHK7 | TNN | 124.9 | 131.4 | 138.9 | 66.5 | 67.1 | 71.3 | 1.249E-04 | 1.929 |
| A0A0D9RRF5 | STARD3NL | 133.7 | 127.2 | 126.0 | 76.5 | 69.6 | 67.1 | 9.644E-05 | 1.815 |
| A0A0D9RYT2 | SUCLA2 | 123.6 | 117.9 | 120.4 | 73.1 | 80.7 | 84.3 | 3.641E-04 | 1.520 |
| A0A0D9SA04 | ACTRT1 | 122.3 | 122.9 | 120.8 | 77.8 | 79.4 | 76.9 | 1.368E-06 | 1.563 |
| A0A0D9QVL6 | MANBA | 128.9 | 142.3 | 114.4 | 71.2 | 77.9 | 65.2 | 2.973E-03 | 1.799 |
| A0A0D9S6G4 | / | 118.2 | 128.8 | 120.0 | 76.4 | 84.6 | 72.1 | 8.159E-04 | 1.574 |
| A0A0D9RJQ1 | FRZB | 129.8 | 130.2 | 133.4 | 65.6 | 70.7 | 70.3 | 6.276E-06 | 1.904 |
| A0A0D9S6I0 | CSF1 | 132.8 | 130.2 | 128.7 | 70.5 | 67.6 | 70.2 | 2.228E-06 | 1.880 |
| A0A0D9R525 | GCNT2 | 116.5 | 125.9 | 123.3 | 77.5 | 77.1 | 79.8 | 1.164E-04 | 1.560 |
| A0A0D9S0T8 | CENPN | 122.8 | 123.8 | 118.4 | 75.4 | 81.8 | 77.8 | 6.472E-05 | 1.553 |
| A0A0D9RBC5 | PAXBP1 | 123.2 | 117.3 | 122.9 | 83.8 | 79.9 | 73.0 | 3.342E-04 | 1.535 |
| A0A0D9R938 | / | 118.5 | 120.0 | 123.1 | 78.2 | 75.3 | 84.9 | 1.995E-04 | 1.517 |
| A0A0D9R3D2 | NSRP1 | 119.5 | 127.8 | 122.8 | 77.4 | 73.7 | 78.7 | 7.961E-05 | 1.611 |
| A0A0D9RES4 | CXCL12 | 131.5 | 117.9 | 135.9 | 74.5 | 73.3 | 67.0 | 6.477E-04 | 1.794 |
| A0A0D9RNM5 | LOXL1 | 139.8 | 155.5 | 143.3 | 55.1 | 52.8 | 53.5 | 4.317E-05 | 2.717 |
| A0A0D9RYB2 | / | 131.7 | 145.3 | 129.1 | 58.5 | 65.9 | 69.5 | 2.917E-04 | 2.094 |
| A0A0D9S7S7 | MAP7D1 | 124.2 | 122.0 | 120.4 | 81.0 | 78.5 | 73.9 | 4.645E-05 | 1.571 |
| A0A0D9RXD1 | MGAT4A | 118.9 | 123.0 | 118.6 | 82.9 | 77.1 | 79.4 | 5.228E-05 | 1.506 |
| A0A0D9S0L2 | BTNL9 | 124.8 | 132.9 | 128.2 | 70.6 | 72.5 | 71.1 | 1.883E-05 | 1.802 |
| A0A0D9RR59 | NFU1 | 154.9 | 144.8 | 151.8 | 48.6 | 45.5 | 54.4 | 1.412E-05 | 3.040 |
| A0A0D9RCJ3 | EXOC3L4 | 136.5 | 139.4 | 146.9 | 57.7 | 58.5 | 60.9 | 1.462E-05 | 2.387 |
| A0A0D9RWB0 | CHSY1 | 123.1 | 121.2 | 120.7 | 81.5 | 73.7 | 79.7 | 6.167E-05 | 1.554 |
| A0A0D9S0Q6 | NTN4 | 159.4 | 153.6 | 153.6 | 41.6 | 46.6 | 45.2 | 1.394E-06 | 3.498 |
| A0A0D9S541 | GTF2H3 | 118.5 | 121.5 | 121.1 | 79.5 | 79.6 | 79.8 | 1.729E-06 | 1.512 |
| A0A0D9R526 | DNAH3 | 135.8 | 125.3 | 128.6 | 69.3 | 69.1 | 71.9 | 5.000E-05 | 1.853 |
| A0A0D9RVX5 | INTS10 | 129.4 | 121.2 | 122.6 | 77.4 | 78.1 | 71.3 | 1.259E-04 | 1.646 |
| A0A0D9S6P5 | RWDD3 | 135.8 | 134.0 | 144.6 | 58.0 | 63.4 | 64.1 | 3.631E-05 | 2.234 |
| A0A0D9RWU8 | CAPSL | 166.5 | 161.5 | 148.6 | 38.4 | 47.0 | 38.0 | 4.212E-05 | 3.862 |
| A0A0D9RLF0 | YEATS2 | 122.3 | 118.7 | 119.2 | 76.0 | 82.5 | 81.3 | 6.251E-05 | 1.502 |
| A0A0D9RU79 | TGOLN2 | 138.1 | 136.3 | 126.0 | 64.0 | 61.6 | 73.9 | 2.304E-04 | 2.007 |
| A0A0D9R4S1 | FARS2 | 113.2 | 139.1 | 119.5 | 59.6 | 99.1 | 69.4 | 2.800E-02 | 1.630 |
| A0A0D9RCT6 | / | 122.9 | 125.3 | 122.9 | 74.4 | 78.3 | 76.2 | 4.312E-06 | 1.621 |
| A0A0D9RHB4 | DARS2 | 131.5 | 130.0 | 132.0 | 69.9 | 65.3 | 71.3 | 5.249E-06 | 1.906 |
| A0A0D9S4V7 | B3GNT4 | 137.6 | 119.3 | 127.5 | 61.9 | 76.1 | 77.5 | 1.497E-03 | 1.784 |
| A0A0D9RXE5 | LRP11 | 145.9 | 149.3 | 149.2 | 51.0 | 56.1 | 48.6 | 2.624E-06 | 2.854 |
| A0A0D9S7V5 | AZIN2 | 129.6 | 120.7 | 126.3 | 74.9 | 72.7 | 75.8 | 4.993E-05 | 1.686 |
| A0A0D9QZ31 | GDF11 | 151.3 | 152.2 | 147.4 | 51.1 | 49.3 | 48.7 | 4.232E-07 | 3.024 |
| A0A0D9RXH1 | CASP8AP2 | 145.8 | 129.5 | 145.5 | 54.0 | 64.6 | 60.6 | 2.037E-04 | 2.348 |
| A0A0D9R0E6 | SETD1A | 121.5 | 129.1 | 124.9 | 76.8 | 71.7 | 76.1 | 5.006E-05 | 1.672 |
| A0A0D9R5K7 | INO80 | 125.0 | 122.4 | 115.8 | 76.8 | 75.3 | 84.7 | 4.594E-04 | 1.534 |
| A0A0D9RL22 | TLR4 | 141.5 | 145.6 | 130.5 | 58.1 | 67.1 | 57.2 | 1.413E-04 | 2.289 |
| A0A0D9RW23 | C5orf34 | 121.8 | 124.0 | 115.2 | 83.7 | 77.3 | 78.1 | 2.569E-04 | 1.510 |
| A0A0D9RGL3 | IFT140 | 125.2 | 130.2 | 135.5 | 60.9 | 71.4 | 76.7 | 3.885E-04 | 1.870 |
| A0A0D9SDL7 | B3GNT2 | 139.3 | 137.8 | 138.6 | 60.3 | 60.5 | 63.5 | 2.683E-07 | 2.256 |
| A0A0D9RKT8 | COL28A1 | 123.6 | 122.6 | 131.8 | 70.4 | 76.6 | 75.0 | 1.137E-04 | 1.703 |
| A0A0D9RA98 | BNC2 | 170.0 | 172.4 | 173.6 | 24.3 | 29.5 | 30.3 | 3.026E-07 | 6.136 |
