# Supplementary figures and images for: Altered Proteomic Profile of Exosomes Secreted from Vero Cells Infected with Porcine Epidemic Diarrhea Virus
Source: Viruses. 2023 Jul 27;15(8):1640. doi: 10.3390/v15081640 (PMC10459195; doi:10.3390/v15081640)

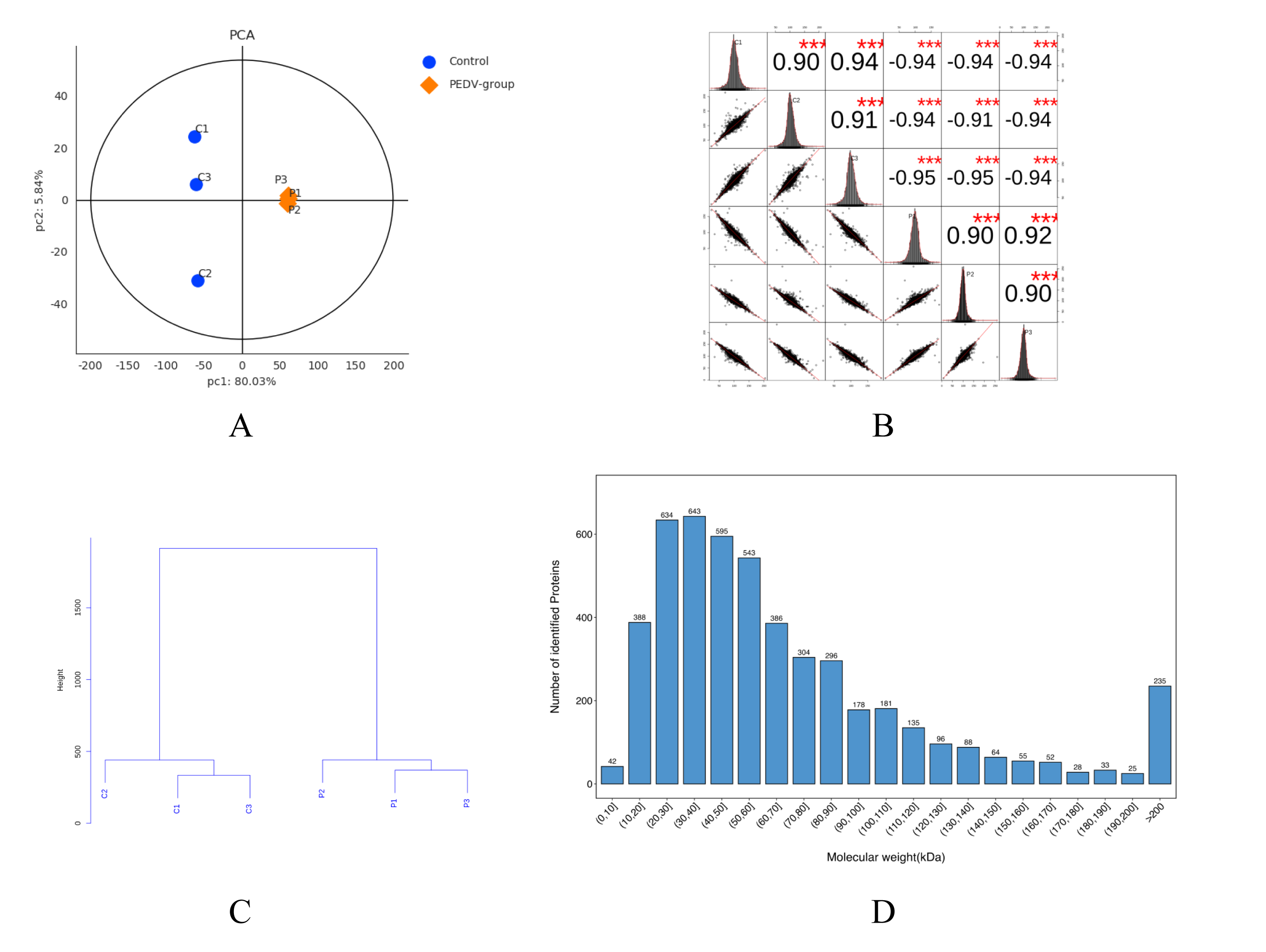

Supplement: Supplementary file 1 [file viruses-15-01640-s001.zip › Supplementary Files/Supplementary Figure S1.tif]
